# Quantitative super-resolution localization microscopy of DNA in situ using Vybrant® DyeCycle™ Violet fluorescent probe

**DOI:** 10.1016/j.dib.2016.01.041

**Published:** 2016-01-29

**Authors:** Dominika Żurek-Biesiada, Aleksander T. Szczurek, Kirti Prakash, Gerrit Best, Giriram K. Mohana, Hyun-Keun Lee, Jean-Yves Roignant, Jurek W. Dobrucki, Christoph Cremer, Udo Birk

**Affiliations:** a Laboratory of Cell Biophysics, Faculty of Biochemistry, Biophysics and Biotechnology, Jagiellonian University, Gronostajowa 7, 30-387 Kraków, Poland; b Institute of Molecular Biology (IMB), Ackermannweg 4, 55128 Mainz, Germany; c Institute for Pharmacy and Molecular Biotechnology (IPMB), University of Heidelberg, Im Neuenheimer Feld 364, D-69120 Heidelberg, Germany; d Kirchhoff Institute for Physics, University of Heidelberg, Heidelberg, Germany; e Department of Physics, University of Mainz (JGU), Staudingerweg 7, 55128 Mainz, Germany

**Keywords:** Super-Resolution, DNA, dSTORM, Localization microscopy, Fluorescence, Chromatin, Vybrant violet, DNA dye, Single molecules, Nucleus

## Abstract

Single Molecule Localization Microscopy (SMLM) is a recently emerged optical imaging method that was shown to achieve a resolution in the order of tens of nanometers in intact cells. Novel high resolution imaging methods might be crucial for understanding of how the chromatin, a complex of DNA and proteins, is arranged in the eukaryotic cell nucleus. Such an approach utilizing switching of a fluorescent, DNA-binding dye Vybrant® DyeCycle™ Violet has been previously demonstrated by us (Żurek-Biesiada et al., 2015) [1]. Here we provide quantitative information on the influence of the chemical environment on the behavior of the dye, discuss the variability in the DNA-associated signal density, and demonstrate direct proof of enhanced structural resolution. Furthermore, we compare different visualization approaches. Finally, we describe various opportunities of multicolor DNA/SMLM imaging in eukaryotic cell nuclei.

## Specifications table

**Table:** 

Subject area	*Biology*
More specific subject area	*Super-resolution microscopy of cell nuclei.*
Type of data	*Tables, Figures*
How data was acquired	*Super-resolution localization microscopy data based on blinking of* Vybrant® DyeCycle™ Violet *dye with an affinity to nuclear DNA; 2D data constitute an optical slice (<500 *nm *thickness) through cell nuclei.*
Data format	*Analyzed SMLM list of positions and reconstructions.*
Experimental factors	*VERO-B4, HL-1or MSU 1.1 cell nuclei stained with the dye at a variety of concentrations and chemical environments.*
Experimental features	*Blinking of the green-emitting form of the dye induced by 491 *nm *laser of high intensity.*
Data source location	*Mainz, Germany; Krakow, Poland*
Data accessibility	*The data are with this article.*

## Value of the data

• Single molecule localization microscopy of DNA based on blinking of the photoproduct is possible with single wavelength excitation yielding improved structural resolution.
• A number of other fluorescent probes perform well in the imaging conditions used for this DNA dye, thus enabling multicolor single molecule localization microscopy of DNA and, for instance, immunofluorescently labeled targets.
• Quantitative evaluation of super-resolution DNA images.

## 1 Data

Single molecule localization microscopy reconstructions of nuclear DNA stained with the fluorescent dye Vybrant® DyeCycle™ Violet.

## 2 Experimental design, materials and methods

Cells on coverslips were fixed and stained with various concentrations using Vybrant® DyeCycle™ Violet (VdcV). Subsequently they were embedded in an imaging buffer of interest and Single Molecule Localization Microscopy (SMLM) images were acquired using a microscope in a widefield mode. Single molecule signals associated with DNA in the cell nucleus were acquired. Their exact locations constitute the basis for reconstruction of high resolution images.

### 2.1 Optimization of imaging buffers for SMLM of Vybrant® DyeCycle™ Violet

In search for optimal imaging conditions for SMLM, potentially in combination with Structured Illumination Microscopy (SIM), a number of imaging buffers with various components were tested, including: PBS, glycerol, mercaptoethylamine (MEA), glucose oxidase in combination with catalase and glucose, ascorbic acid and Prolong Gold®. All of these factors may influence the number of fluorescent cycles (i.e. photons emitted by a single molecule) during an ‘ON’-state. Note that the number of emitted photons per fluorescent molecule (*N*) directly influences localization precision, the latter being proportional to N^−1/2^. The observed large differences in the number of signals detected from the green-emitting form of Vybrant® DyeCycle™ Violet depends on the concentration of the oxygen scavenging system, i.e. on the oxygen content. As reported [2], oxygen is the primary source of irreversible damage inflicted upon the fluorophore, especially upon high intensity illumination; such permanent photobleaching results in a deterioration of the image quality (photon count, density of molecule signals).

**Fig. 1:**
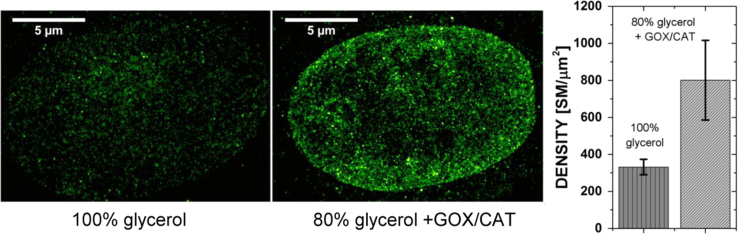
The influence of the enzymatic oxygen scavenging system (abbreviated as GOX/CAT), diluted in PBS and added to glycerol, on VdcV SMLM image quality. Under the conditions used in Fig. 1, glycerol alone provided an average density of about 300 single molecule (SM) signals per µm^2^, or 17.3×17.3 SM/µm^2^, i.e. one signal per 1000/17 nm=59 nm, corresponding to an average estimated structural resolution of ~2×59 nm. However, by adding the specifically designed enzymatic oxygen scavenging medium, the average density of SM signals was increased several times, resulting in an SMLM image with further enhanced structural resolution (based on the same number of image frames). For a quantitative comparison we used only VdcV at 10 nM, i.e. at a concentration where saturation of the detector does not occur at the beginning of the illumination procedure. This protocol made it possible to run a measurement without prior bleaching. Using an excitation wavelength of $\lambda_{\mathrm{exc}}$=491 nm (illumination intensity 0.3 kW/cm^2^) and fluorescence emission registration in the range of 585–675 nm, 20,000 frames were collected. Signal densities obtained in the images of the cell nuclei: 207 SM/µm^2^ in 100% glycerol (*n*=7) and 679 SM/µm^2^ in the imaging buffer (*n*=9; *n*=number of cells analyzed). 207 SM/µm^2^ (=14.4×14.4 SM µm^2^) corresponds to the next neighbor distance of one signal per 1000/14.4 nm=70 nm, resulting in an average estimated structural resolution of ~2×70 nm; 679 SM/µm^2^ (=26×26 SM µm^2^) corresponds to the next neighbor distance of one signal per 1000/26 nm=38 nm, resulting in an average estimated structural resolution of ~2×38 nm. Note that the optical (two point) resolution depends on the precision of localization, whereas the structural resolution depends also on the density of signals, thus it may be different for different SMLM images, even if the localization precision remains the same.

All of the buffers mentioned above were tested in combination with various concentrations of VdcV (Table 1). The most prominent and efficient blinking of the green-emitting form of VdcV was observed when an enzymatic oxygen scavenging system, dissolved in glycerol, was used (Fig. 1).

### 2.2 Conversion of Vybrant® DyeCycle™ Violet to its green-emitting form

The localization microscopy based on VdcV reported here shares some similarities with the results reported for SMLM imaging of Hoechst 33258, Hoechst 33342 and DAPI [3]. In our recent publications [11], [12] we described the properties of these dyes using mass spectrometry and found a significant dependence of the abundances of the protonated forms of Hoechst 33258 on pH and the presence of hydrogen peroxide. A similar blinking behavior of Hoechst dyes and VdcV, and the fact that we used the same imaging buffers to induce blinking, suggest that the photophysical mechanism underlying the conversion of the blue-emitting to the green-emitting form of VdcV may be protonation, as it was demonstrated for bisbenzimide dyes [11]. Further studies are required to understand the photophysics of VdcV on the single molecule level in the context of the chemical environment.

From a general methodological point of view it may be noted that the SMLM method used here largely simplifies the approach to super-resolution microscopy, as it requires only monochromatic illumination for both photo-switching and fluorescence read-out; standard (or slightly modified) sample preparation; and a single type of organic fluorophores. Previously, such experimental approach has been successfully applied in other cases, such as Alexa and Atto dyes and green fluorescent proteins [6], [13]. In this report, it is shown that a very similar approach can be effectively used to achieve SMLM imaging of nuclear and chromosomal DNA distribution directly stained with VdcV, as the single laser wavelength not only induces blinking, but it may also be used to induce photoconversion, resulting in a red-shift of the emission wavelength.

### 2.3 Single molecule fluorescent bursts – raw data

**Fig. 2:**
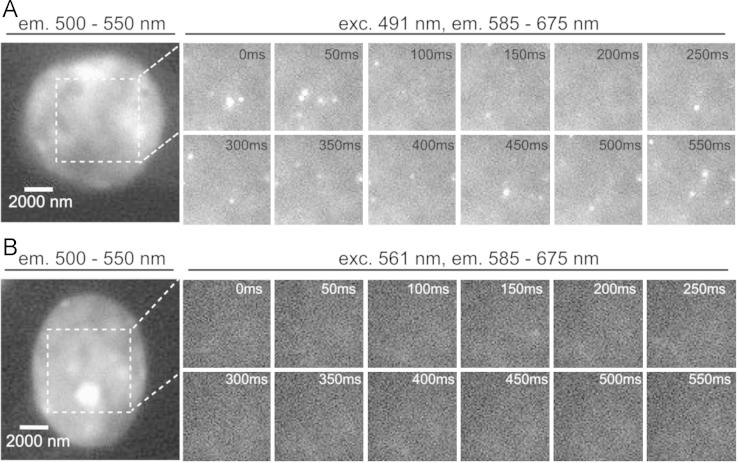
Single molecule fluorescent bursts in cells stained with Vybrant® DyeCycle™ Violet detected in the green-yellow emission range (585–675 nm) using high intensity single wavelength excitation (0.525 kW/cm^2^, $\lambda_{\mathrm{exc}}$=491 nm) after several minutes of illumination (A). Molecules of the green-emitting form of VdcV (occurring naturally under standard conditions) were reversibly bleached and reappeared stochastically in the green–yellow detection channel. Concentration of VdcV was 500 nM. Note that the relative time values given may actually slightly differ from the real ones since the read-out time of the camera is not taken into account. (B) Single molecule localization acquisition in the same detection channel using high excitation intensity 561 nm illumination (appropriate for blinking of Alexa555 or Alexa568), in the same detection channel as the second reporter molecule, distinguished on the basis of distinct excitation spectra. As can be inferred from the raw data images, blinking induced by 561 nm excitation is negligible in the absence of Alexa reporter molecules. However, we noted that some blinking did appear and was mostly associated with the cytoplasm.

Single molecule fluorescent signals of DNA-bound Vybrant® DyeCycle™ Violet were acquired under 491 nm and 561 nm excitation wavelength (Fig. 2).

### 2.4 Experimental measurement of image resolution in VdcV/SMLM data sets

**Fig. 3:**
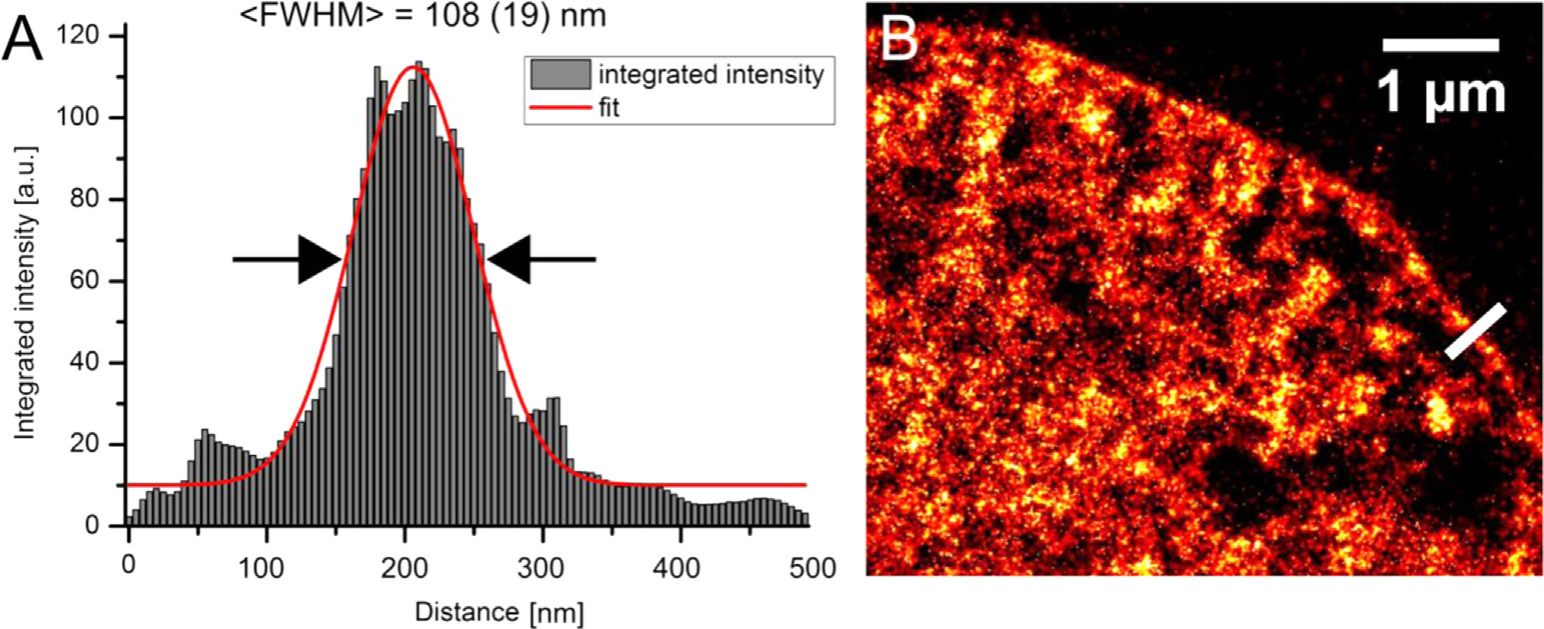
Sub-diffraction measurement of heterochromatin aligned to the nuclear envelope in HL-1 cells. The thickness of this chromatin structure is known to be low, and the structure highly compacted. It has been measured after fitting a Gaussian function and equals 108(±19)  nm (*n*=5). Integration has been performed in 100 nm×500 nm rectangle regions. (A) An example of an intensity profile across a heterochromatin region (as highlighted in B by the white line) at the nuclear membrane. (B) A region of the nucleus in which the measurement has been performed.

**Fig. 4:**
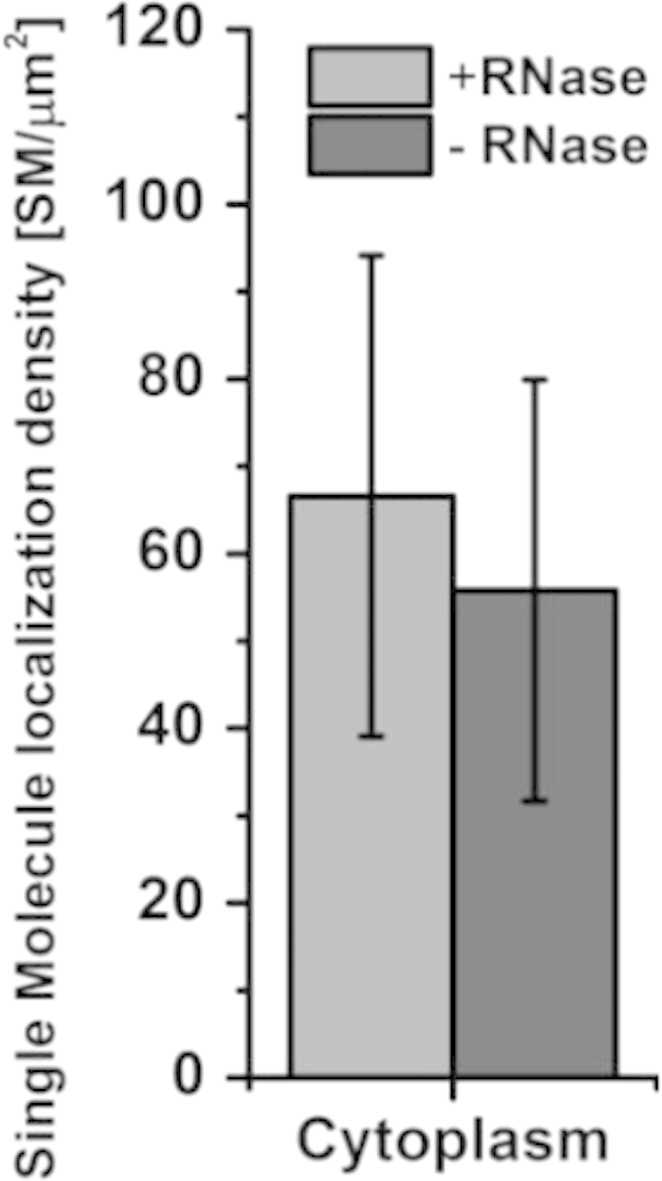
Single Molecule Localization data acquired in the cytoplasm. 1 h RNase treatment at 37 °C does not influence the density of single molecule localizations in the cytoplasm indicating that contribution to the total signal arising from VdcV bound to RNA was negligible (*n*=3).

Image resolution was estimated by extracting a line plot across distinct structures such as heterochromatin regions at the nuclear envelope (see Fig. 3). Alternatively, it would be possible to extract Fourier-Ring-Correlation (FRC) values from the whole nucleus. This has been done previously for Vybrant Violet stained HL-1 cell nuclei [16], with very similar values as the ones obtained here. Furthermore, we analyzed the possible contribution of VdcV bound to RNA (see Fig. 4) by comparing the signal density in the cytoplasm with/without RNase added. Since the values extracted with/without RNase added are almost the same, the contribution of VdcV bound to RNA is assumed to be insignificant. Compared with the high density regions in the nucleus, the signal density in the cytoplasm was about two orders of magnitude lower (see Section 2.7).

### 2.5 An influence of hydrogen peroxide and acidic environment on VdcV photoproduct formation

**Fig. 5:**
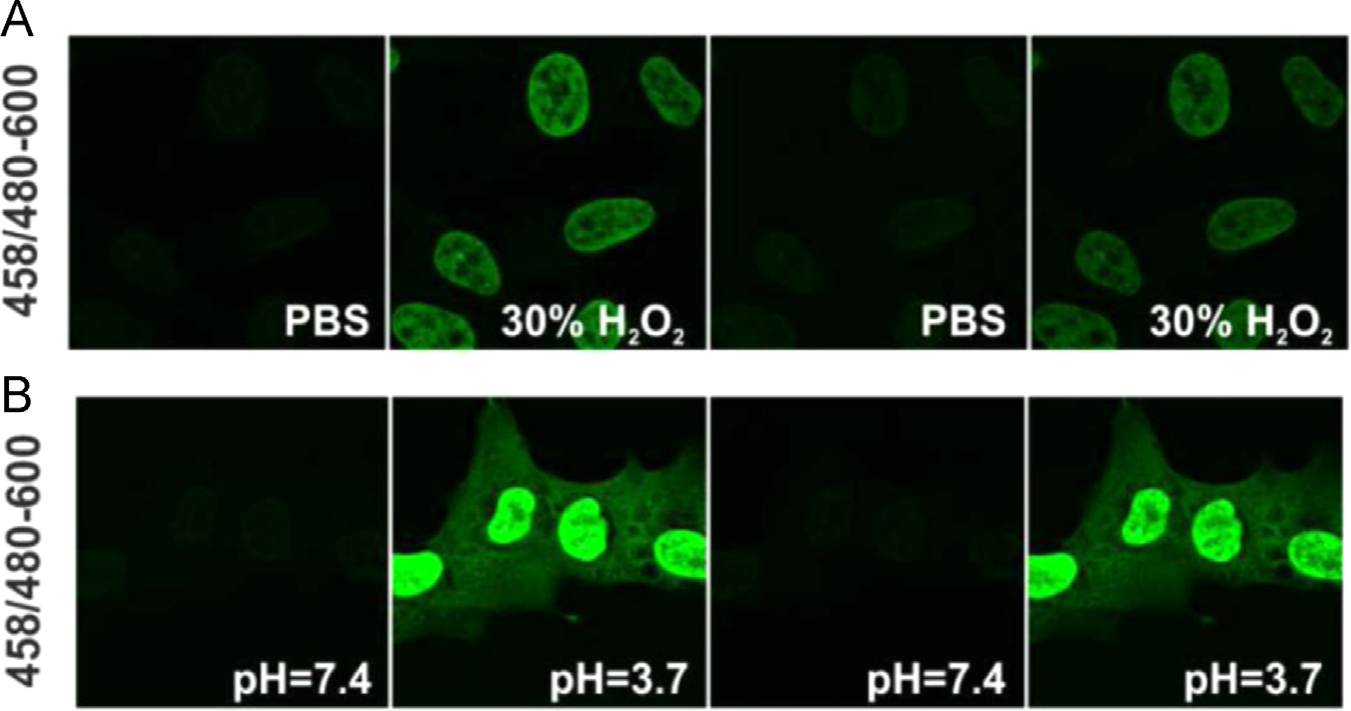
Vybrant® DyeCycle™ Violet fluorescence properties are altered by low pH or a high concentration of hydrogen peroxide. MSU 1.1 cells were stained with VdcV at 1 µM, green emission of the dye excited by 458 nm was detected in the 480–600 nm band, and the chemical environment was exchanged as stated in the image panels. Images presented here were acquired using constant excitation intensity, and were acquired sequentially within minutes. This behavior of VdcV strongly resembles DAPI and Hoechst dyes, as we previously reported [11]. Note that the changes reported here are reversible. The experimental procedures were reported previously [11]. The binding mode of VdcV is not disclosed, however its properties resemble minor-groove binders as Hoechst dyes.

Fixed and permeabilized cells were stained with Vybrant Violet and imaged on confocal microscope when immersed with PBS, 30% hydrogen peroxide or buffer of pH=3.7. Fluorescence in the green region of the emission spectrum was collected. (Fig. 5).

### 2.6 Localization data thresholding for estimation of multiple blinking

**Fig. 6:**
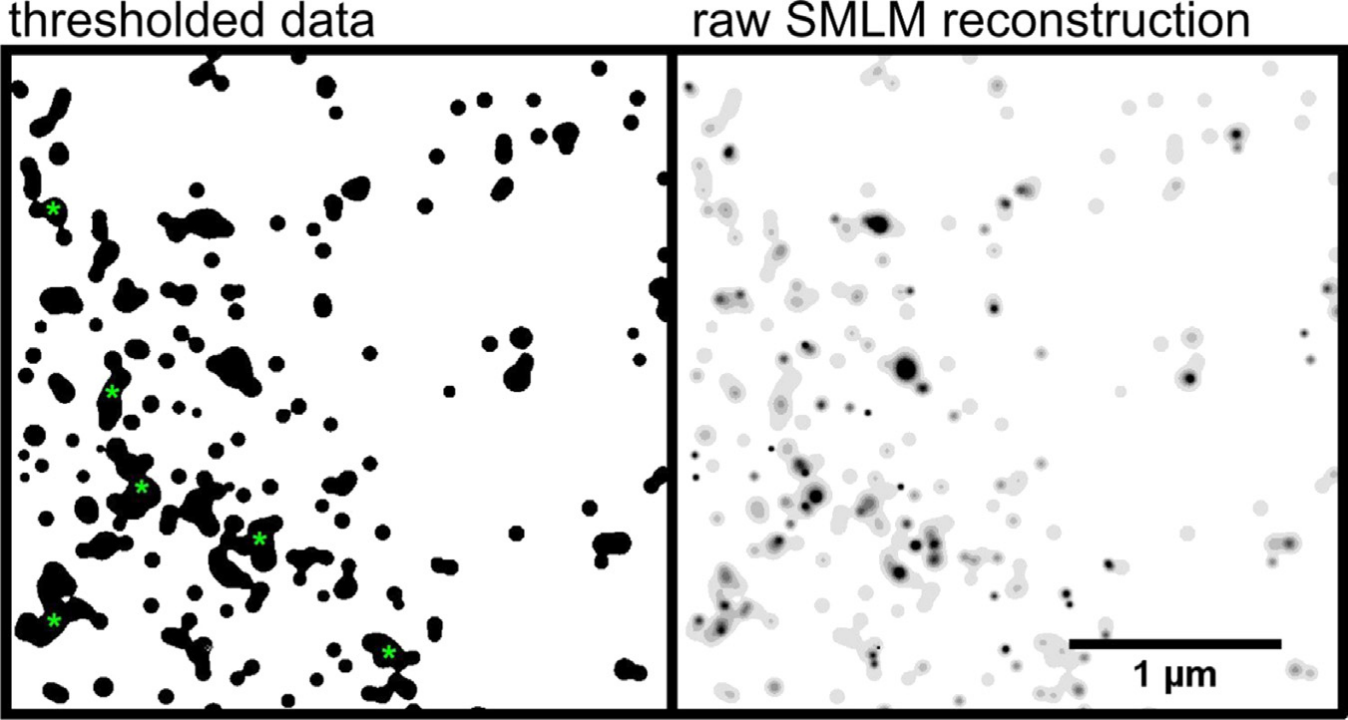
In order to provide an estimate of the number of re-appearing single molecule events in our SMLM images the cytoplasm containing mitochondrial DNA and RNA was analyzed. First the SMLM image of VdcV-stained cells was acquired (30,000 frames, 50 ms exposure time). Then the reconstructed raw SMLM image (example on the right) was thresholded in order to include all pixel values >0 to the binary mask (left). Next the number of objects in the binary mask was calculated and the total number of single molecule localizations (SM) was attributed to the number of binary objects. The number of objects in the binary mask amounted to 13.8±1.2 SM/µm^2^, which is more than the number of theoretical Airy discs fitting in an area of a square micron (roughly 3). This way we obtained a value of 4.1±1.2 SM per cluster (*n*=3) which constitutes a measure of multiple blinking in our SMLM images. Commonly, clearly overlapping single molecule signals are present in such analysis (indicated with green asterisks) and they will contribute to overestimating the number of single molecule localizations per single molecule of VdcV. Thus, the real measure of multiple blinking falls below 4 times per molecule throughout an acquisition.

In order to estimate how many times on average a single VdcV molecule appears as a fluorescent signal throughout the acquisition, a localization microscopy was performed on the cellular cytoplasm where relatively small amount of RNA and mitochondrial DNA are present. The dataset was visualized using the method based on blurring single molecule positions with the respective localization precision. Subsequently, the reconstructed 8-bit visualization was converted into a binary image by thresholding intensities above 0. The number of detected objects in the binary image was compared with the number of single molecule localizations. (Fig. 6).

### 2.7 Dependence of the number of single molecule bursts, detected in a single excitation experiment, on the concentration of VdcV and the length of image acquisition

**Fig. 7:**
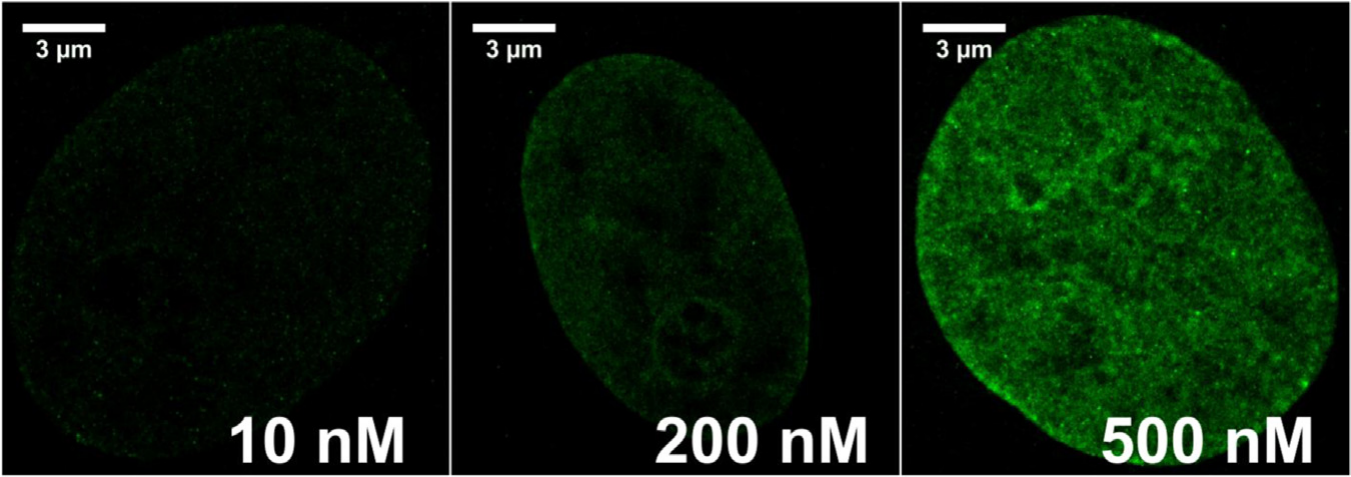
Different concentrations of VdcV bound to nuclear DNA influence the signal density in the reconstructed images. Images of Vero-B4 nuclei were acquired using SMLM with high intensity 491 nm illumination (0.525 kW/cm^2^) for three concentrations of VdcV (10 nM, 200 nM, and 500 nM). We observed the following average signal densities in the detection channel ($\lambda_{\mathrm{em}}$: 585–675 nm): 944 SM/µm^2^ (10 nM), 1735 SM/µm^2^ (200 nM) and 4818 SM/µm^2^ (500 nM). 20,000 frames were recorded in each measurement.

We tested various concentrations of VdcV, ranging from 10 to 1000 nM. Using a 20,000 frame protocol and a 491 nm excitation, we detected very high numbers of fluorescent bursts that increased with the increasing concentration of the dye. Differences in the range of 300–1000 nM VdcV were not significant. Examples of SMLM images acquired using different concentrations of VdcV are shown in Fig. 7.

**Fig. 8:**
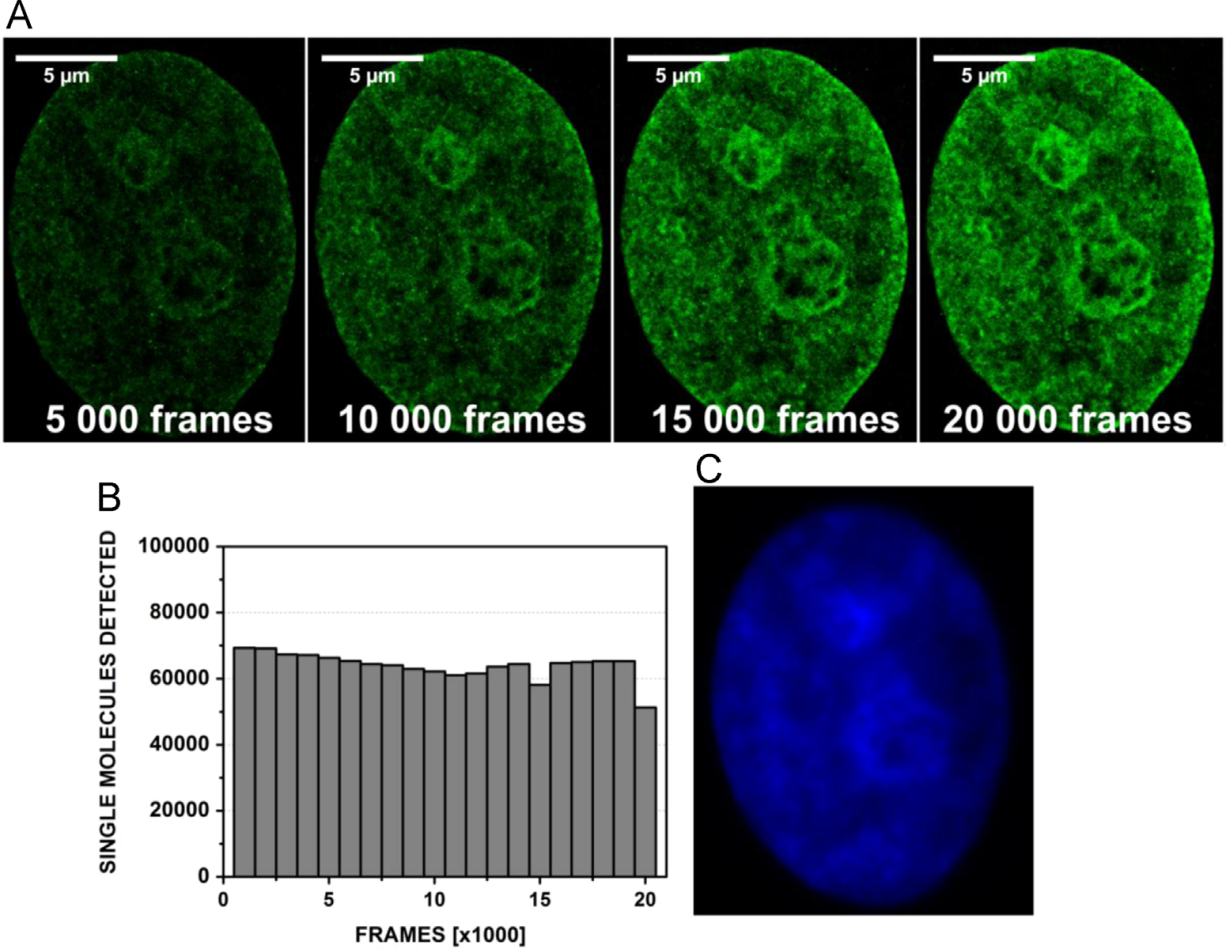
Dependence of the SMLM image quality on the duration of the image acquisition. (A) Reconstructed images of a Vero-B4 cell nucleus stained with 500 nM Vybrant® DyeCycle™ Violet. The images show that the total number of single molecule fluorescent bursts detected depends strongly on the total number of the image frames, i.e. on the length of the image acquisition. Average signal densities for the images: 1496 SM/µm^2^ (5000 frames); 2901 SM/µm^2^ (10,000 frames), 4285 SM/µm^2^ (15,000 frames), 5716 SM/µm^2^ (20,000 frames). SM signals per frame (in the following denoted by *α*): 5000 frames: *α*=1496/5000=0.299 SM/frame; 10,000 frames: *α*=0.290; 15,000 frames: *α*=0.286; 20,000 frames: *α*=0.286; $\lambda_{\mathrm{exc}}$=491 nm (0.525 kW/cm^2^), $\lambda_{\mathrm{em}}$: 585–675 nm. (B) A dependence of the number of the single molecule signals detected per 1000 acquired frames on acquisition time. A slight permanent bleaching (exponential decay) is observed. (C) A widefield image of fluorescence of VdcV in the same Vero-B4 cell nucleus; $\lambda_{\mathrm{exc}}$= 405 nm, $\lambda_{\mathrm{em}}$= 440–490 nm.

Single molecule localization images were reconstructed every 5000 frames. The total number of single fluorescent bursts detected depends almost linearly on the number of frames acquired (Fig. 8), irrespective of the local signal density in the nuclear sample. The exponential decay, i.e. the irreversible photobleaching, amounts to less than 6% for the 20,000 frames acquired (least squares regression). Fig. 8 demonstrates that it is possible to acquire a satisfactory SMLM image within 5000–10,000 frames (total acquisition time approximately 8 min) yielding structural details that cannot be obtained by standard widefield microscopy.

### 2.8 Localization precision and photon counts in SMLM measurements of DNA-bound VdcV in the cell nucleus

**Fig. 9:**
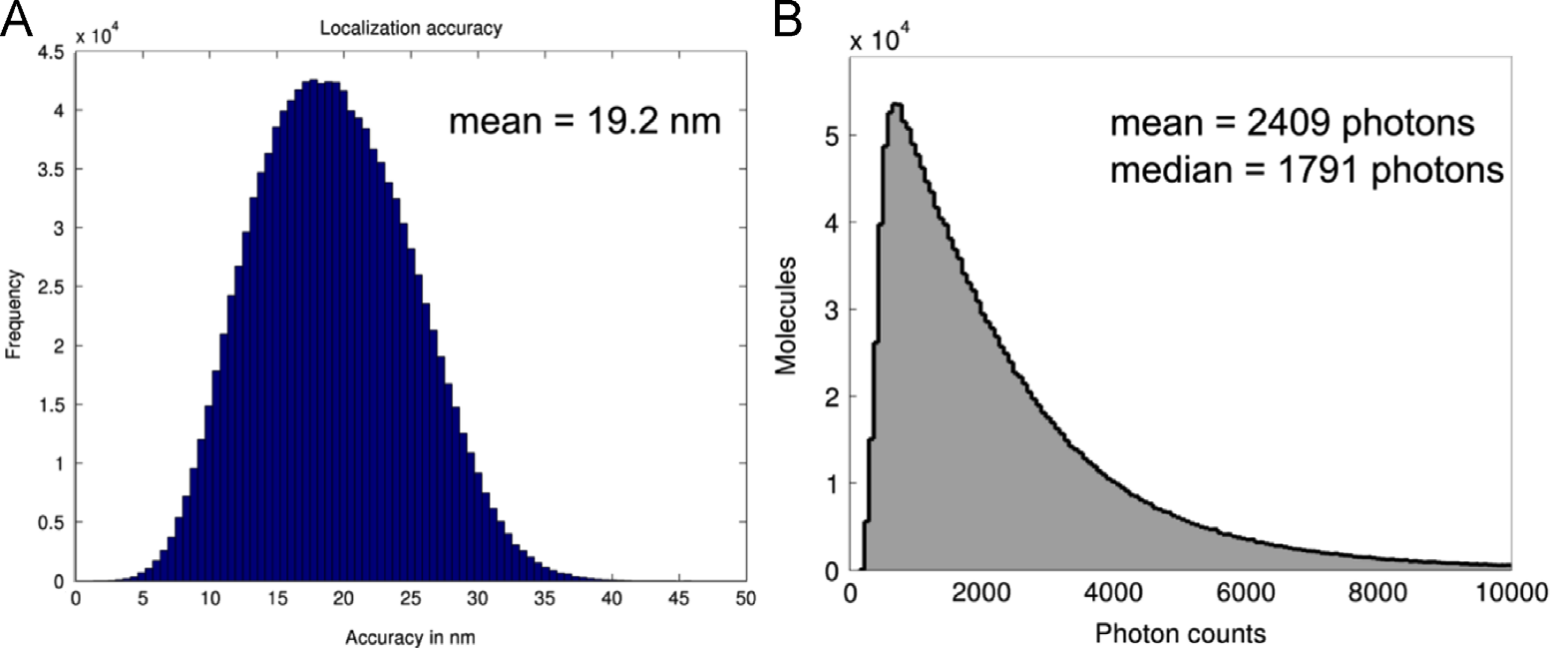
Distribution of the values of localization precision (A) and photon counts (B) obtained for measurements of DNA-bound VdcV in the cell nucleus. (A) Histogram of individual localization precision. The ordinate gives the frequency (number) of individual molecule signals in the cell nucleus evaluated with a given localization precision (abscissa). (B) Histogram of photon counts: the ordinate gives the number of molecules with a detected fluorescence photon count (abscissa).

Fig. 9 depicts statistics of individual signals extracted after acquiring photoconverted DNA-bound VdcV as visualized in Fig. 2 in [1].

### 2.9 Visualization of SMLM measurements of DNA-bound VdcV in the cell nucleus

**Fig. 10:**
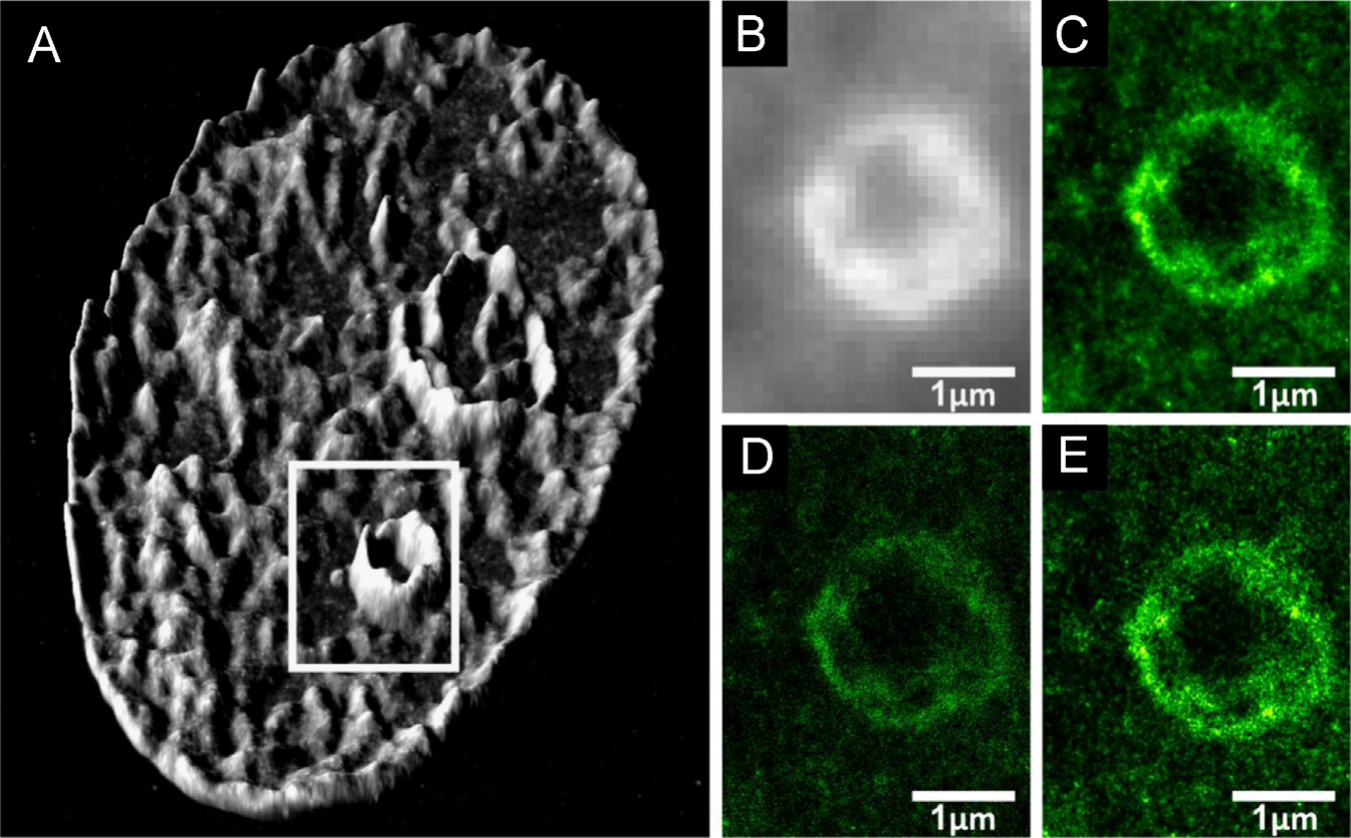
Different methods of presenting high-resolution localization microscopy data and a comparison with a widefield image. (A) Perinucleolar chromatin stained with VdcV and imaged with SMLM. Height (the 3rd dimension) designates the density of the signal (the number of single molecules/µm^2^) of the reconstructed image in Fig. 2 in [1]. The SMLM image was produced after smoothing. (B) A widefield image of the region marked in A. (C) Data points blurred with the respective localization precision. (D) Point representation of single molecule positions. (E) Triangulation originally described by [14] run over the single molecule data set. $\lambda_{\mathrm{exc}}$=491 nm, 0.525 kW/cm^2^, and $\lambda_{\mathrm{em}}$: 585-675 nm.

In Fig. 10, different visual representations of SMLM data, i.e. of signals extracted after acquiring photoconverted DNA-bound VdcV are shown, for the dataset which was visualized in Fig. 2 in [1].

### 2.10 DNA density variability in SMLM images of VdcV

**Fig. 11:**
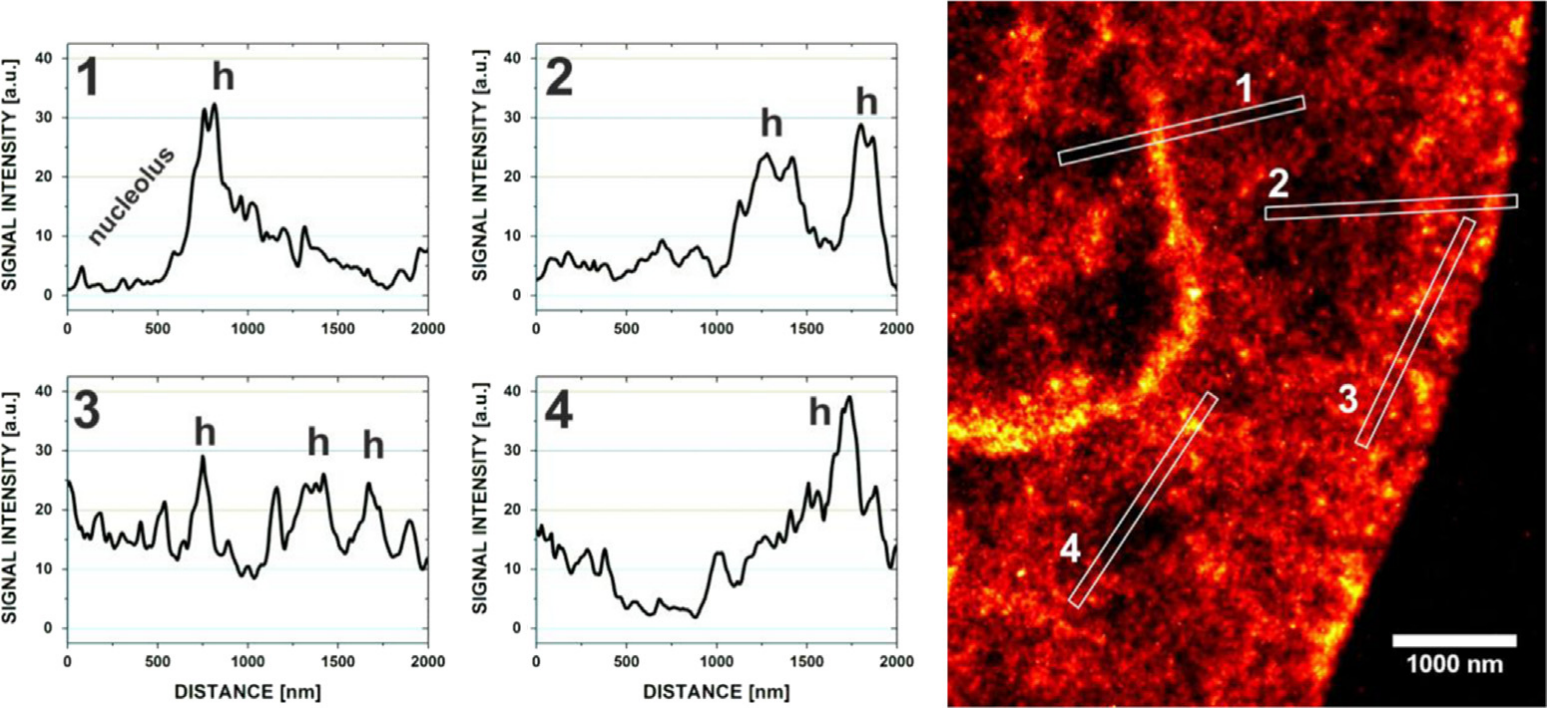
Plot profiles 1 to 4 (left, center) indicating signal density differences occurring on the nanoscale in the reconstructed image of a Vero-B4 cell nucleus (right) stained with 500 nM Vybrant® DyeCycle Violet. The regions most likely representing heterochromatin are marked in the plot profiles with ׳h׳. Plot profiles 1–4 were obtained by integrating the signals over rectangular regions 2 µm long and 100 nm wide. The graphs show the signal density profile in: perinucleolar heterochromatin (1); euchromatin and heterochromatin across the nuclear periphery (2); heterochromatin and euchromatin in the vicinity of the nuclear envelope (3); the lowest DNA density region in the inner region of the nucleus (4).

**Fig. 12:**
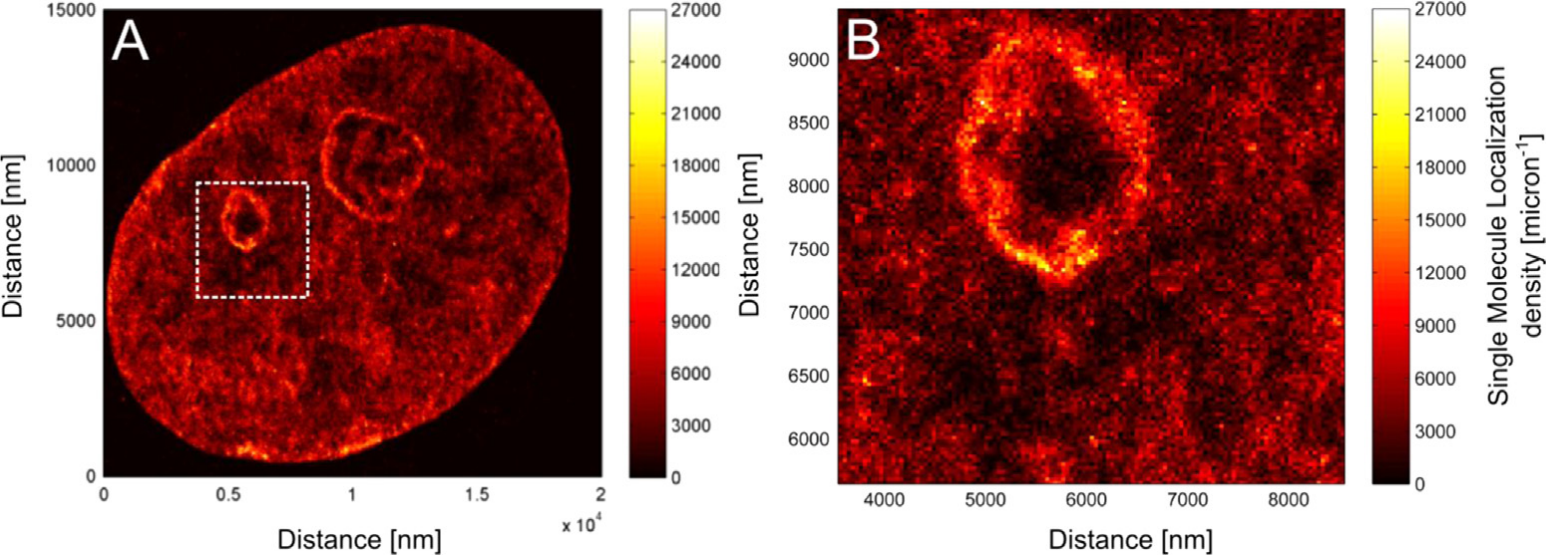
DNA density is visualized after binning the single molecule localizations into a 40 nm x 40 nm grid of pixels. The density presented here is given as the number of localizations per square micron. As can be seen from the color-coded scale, the density of localizations within the image varies by a factor ~20 indicating the existence of DNA-poor areas in the cell nucleus, likely attributable to the Interchromatin Compartments (IC) [15]. Note the low signal of VdcV inside the nucleoli.

In Fig. 11, Fig. 12 the data set visualized in Fig. 2 [1] was used.

### 2.11 An influence of the 405 nm photoconverting illumination on VdcV fluorescence bursts

**Fig. 13:**
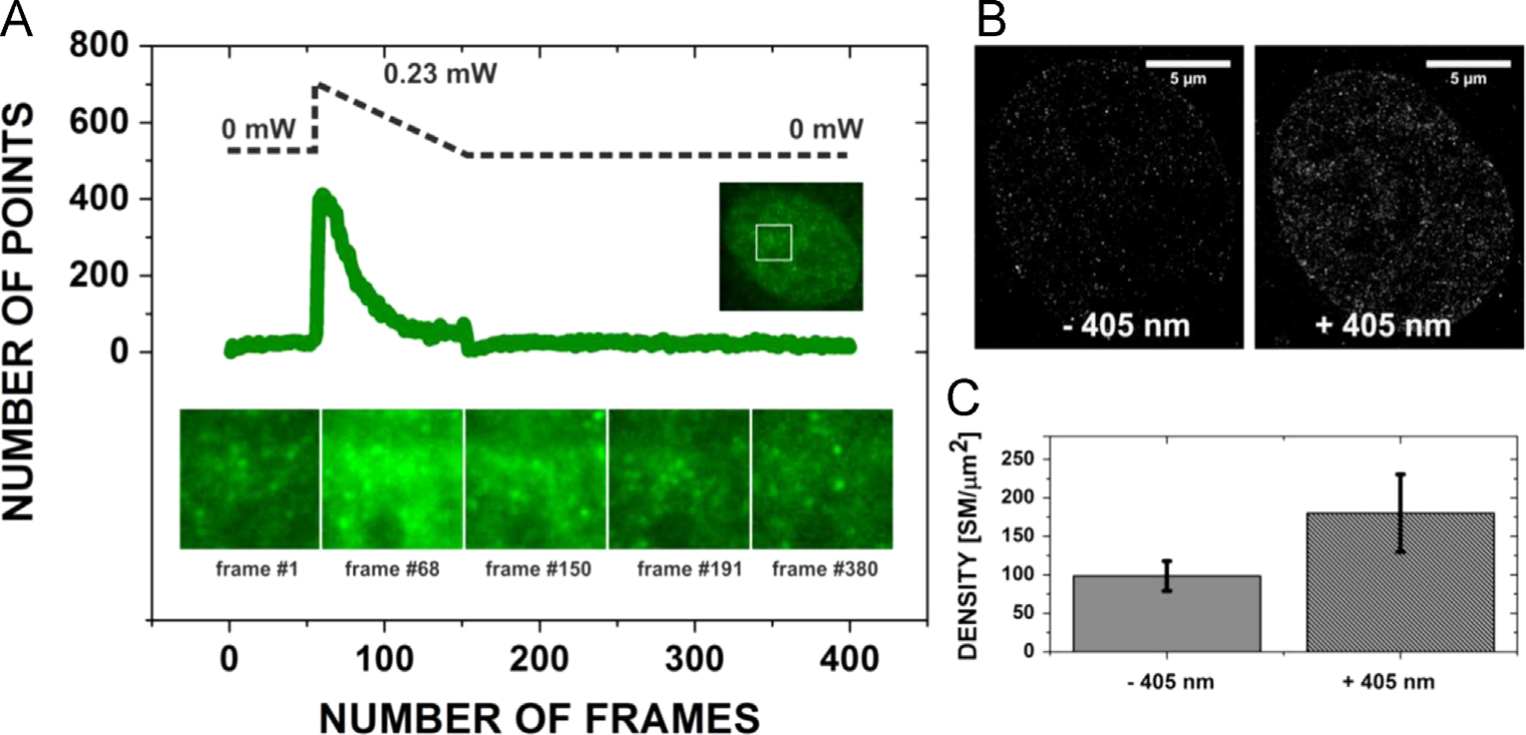
The influence of 405 nm light illumination on SMLM measurements of the green-emitting form of VdcV (150 nM). (A) When 405 nm was applied to the sample, which was already being illuminated with 491 nm excitation, an immediate increase in the number of single molecule localizations detected per frame was detected (frames: 50–200). $\lambda_{\mathrm{exc}}$.=491 nm, 0.525 kW/cm^2^, and $\lambda_{\mathrm{em}}$: 585–675 nm. (B) Examples of SMLM measurements of cells stained with 50 nM VdcV (low concentration) in the presence or absence of 405 nm light (110 µW). When this concentration of VdcV was used, no pre-bleaching was required. Therefore, it was feasible to assess the influence of the photoconverting 405 nm illumination on the number of molecule signals detected. 5000 frames were collected. $\lambda_{\mathrm{exc}}$=491 nm, 0.4 kW/cm^2^, and $\lambda_{\mathrm{em}}$: 585–675 nm. (C) A graph presenting the density of single molecule fluorescent bursts detected under both conditions (abbreviated as SM). (−) Without 405 nm excitation; (+) with additional 405 excitation. Each bar presents an average (and standard deviation) from five experiments.

In Fig. 13, the effects of additional 405 nm illumination on the performance of SMLM measurements are shown. The analysis was performed using the same dataset as visualized in Fig. 3 in [1].

In order to investigate the influence of a low intensity 405 nm illumination on SMLM measurements, namely on the appearance of single molecule fluorescent bursts, we stained Vero-B4 cells with VdcV at a very low concentration (50 nM), allowing for an immediate beginning of the measurement rather than performing the step of pre-bleaching of the excessive amount of the fluorescence signal prior to the measurement. Single molecules of the green-emitting form of VdcV were easily isolated from the relatively low background immediately after applying 1.2 kW/cm^2^ of 491 nm excitation. However, when we applied a linearly decaying ramp of 405 nm laser illumination with a very low initial illumination intensity of 2 W/cm² and a duration (decay time) of approx. 1 min, a significantly higher number of single molecule fluorescence bursts was detected, with a corresponding higher number of the simultaneously fluorescing molecules from the same focal plane (see Fig. 13).

### 2.12 Resolution of the SMLM System, when applied to 2-dimensional structures

**Fig. 14:**
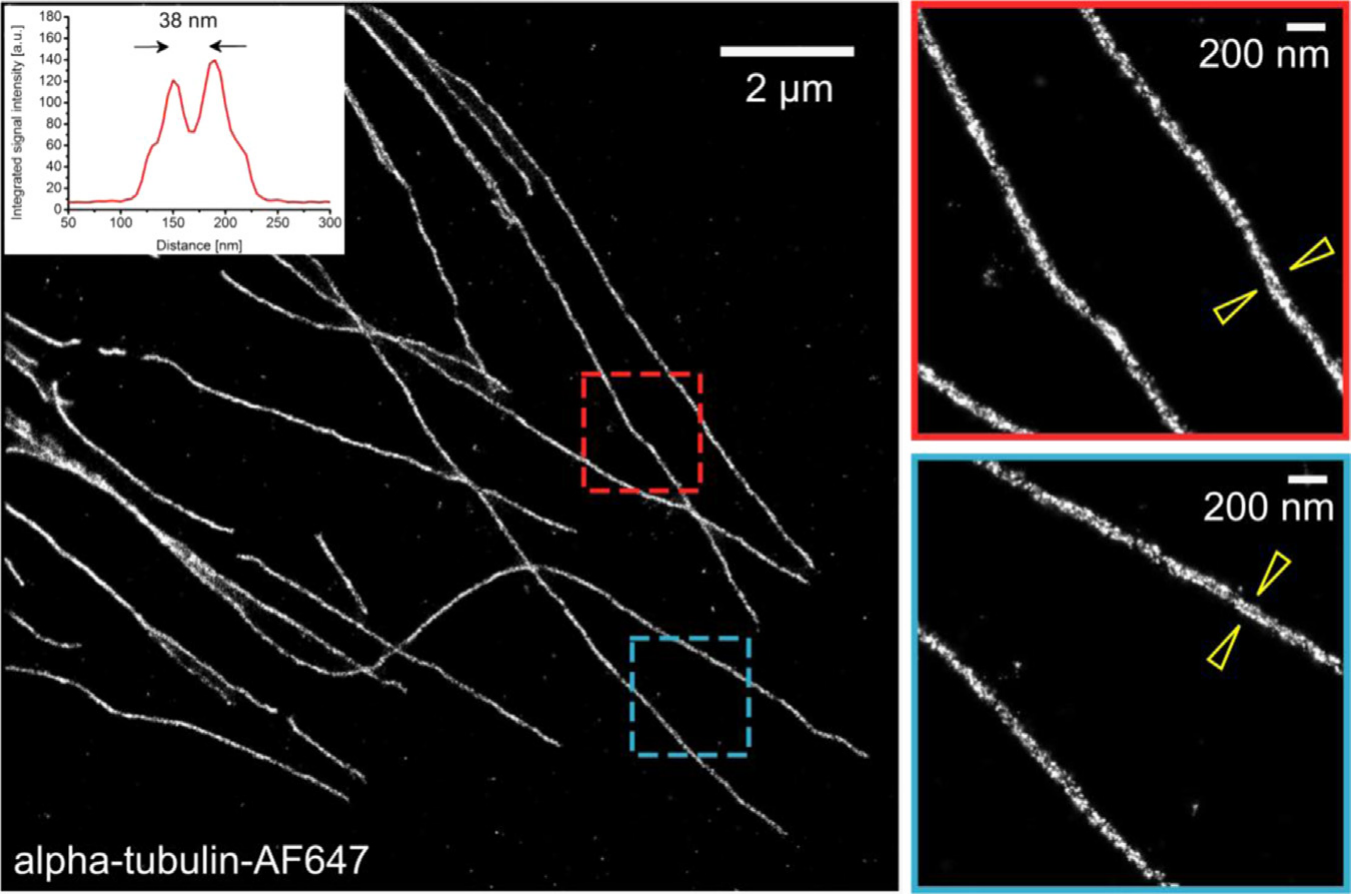
SMLM microscope calibration with a standard specimen of immunofluorescently labeled α-tubulin. Blinking of Alexa 647 (which was conjugated to the secondary antibody) was induced in the presence of primary thiol-containing imaging buffer devoid of oxygen. Reconstruction was performed with the software, which was used also for analysis of DNA/SMLM data [1], [3], [16], [17]. The enlarged insets show that the 2D projection of single molecule fluorophore positions reveals the cylindrical structure of labeled microtubule (diameter=25 nm microtubule+10 nm antibodies)-this results in a bimodal distribution of signals along the long axis of the structure (indicated with arrows) .

Most published SMLM implementations have been optimized for analysis of 1D or 2D structures. Measurements of such structures have been reported typically with a precision in the order of 10 nm. In our approach to visualizing chromatin in 3D intact cell nuclei, we have reported a structural resolution in the order of 100 nm, in spite of the microscope setup being able to produce SMLM data with much higher resolution. In Fig. 14, the results of SMLM measurements of the diameter of fluorescently labeled microtubules are shown, indicating that much higher resolution (37 nm) is achievable, under conducive conditions, with the same instrument used in our study of chromatin [1].

### 2.13 Confocal microscopy of DNA stained with VdcV

**Fig. 15:**
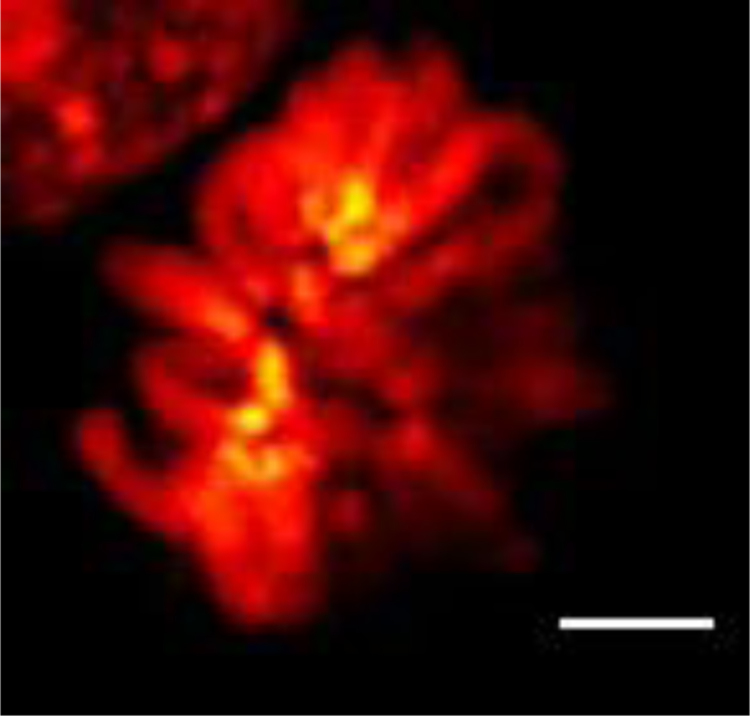
Confocal laser scanning microscopy (CLSM) of Vybrant® DyeCycle™ Violet stained mitotic chromosomes. CLSM is currently the most popular among conventional fluorescence microscopy methods. Scale bar corresponds to 2 µm. No mitotic arrest and hypotonic treatment were applied. Part of an interphase cell nucleus can be seen in the left-top corner of the image.

We tested to what extent we could use VdcV in established confocal laser scanning microscopy (CLSM) to obtain high-resolution images of chromatin structures (see Fig. 15).

## 3 Multicolor DNA/SMLM imaging

A summary/list of fluorescent probes which can be efficiently used in our SMLM approach is shown in Table 2. Particular attention is given to multicolor experiments using fluorescent dyes performing well in conjunction with several DNA dyes suitable for SMLM.

## Figures and Tables

**Table 1:** Qualitative description of various imaging buffers influencing the number of detected molecules of the green-emitting form of VdcV, detected in SMLM imaging of DNA in Vero-B4 nuclei. SNR-signal-to-noise ratio; ׳+׳ observed blinking rate (׳++++׳ is best for high optical and structural resolution SMLM of nuclear DNA); ׳-׳ extent of bleaching during experiments. None of the buffers listed above prevented photoconversion of VdcV.

**No.**	**Imaging buffer**	**Blinking**	**Bleaching**	**Comment**
**1**	**Aqueous PBS**	+	--- -	Single molecule fluorescent bursts have low SNR and are sparse immediately after applying illumination.
**2**	**Glycerol**	++	---	Strong signals at the beginning, relatively fast bleaching, a comparison in Fig. 1.
**3**	**80% glycerol+20% PBS (comprising finally 0.5 mg/ml glucose oxidase, 0.04 mg/ml catalase, 0.1 g/ml glucose)**	++++	-	Buffer used previously for bisbenzimide dyes [3]. For high concentrations of the dye, the CCD detector is easily saturated and the signal bleaches slowly; for low concentrations of the dye the detector is less likely to be saturated, but the signal decreases swiftly due to rapid photobleaching. A successful use of the buffer containing the glucose oxidase-catalase system in PBS for SMLM of some standard fluorophores was reported [4], see Fig. 1.
**4**	**80% glycerol+20% PBS (comprising finally 0.5 mg/ml glucose oxidase, 0.04 mg/ml catalase, 0.1 g/ml glucose)+MEA**	+++	-	Addition of 10 mM MEA to either PBS or glucose oxidase-catalase system was proven to induce blinking in most of the fluorophores covering the entire visible spectrum of fluorescence emission [4] indicating its potential in multicolor experiments. An addition of up to 5 mM MEA (cysteamine) to our optimized buffer did not hamper blinking of VdcV significantly. Higher concentrations reduced blinking strongly.
**5**	**Glycerol+10% PBS**	++	--- -	A high number of signals with moderate SNR was detected at the beginning of the measurement, followed by a rapid decline of blinking events.
**6**	**Prolong Gold**®	+	---	Very low SNR due to high background and low intensity of fluorescent bursts. This standard embedding medium was already reported several times to perform best for AlexaFluor 488 and 594 [5] or standard fluorescent proteins[6], [7].
**7**	**90% glycerol+10% 10 mM ascorbic acid in PBS**	+++	---	High SNR of blinking molecules, fast bleaching disabling further acquisitions after 1-2 min. A similar buffer was proven to be optimal for imaging of PicoGreen-stained DNA [8] and various dyes with specific affinity to several cellular organelles [9].
**8**	**10 mM ascorbic acid in PBS**	+	---	Low SNR of blinking molecules, fast bleaching
**9**	**95% glycerol+5% PBS (comprising finally 0.25 mg/ml glucose oxidase, 0.02 mg/ml catalase, 0.05 g/ml glucose in PBS)**	++++	- -	A low concentration of enzymes performing oxygen scavenging was insufficient to prevent bleaching. This precluded long experiments, i.e. less single molecule fluorescent bursts can be recognized in multiframe acquisitions.
**10**	**PBS+100 mM MEA**	+	--- -	Hardly any blinking of VdcV was observed. This switching buffer used for AlexaFluor dyes was originally reported by [10], later shown to work for a broad spectrum of synthetic dyes [4].

**Table 2:** Qualitative assessment of multicolor SMLM imaging of DNA and other labeled structures. Numerous fluorescent probes were found to perform well in combination with the DNA dyes investigated (number of "+" reflects the quality of performance). For dyes marked with an asterisk it was found that very low intensity illumination at the second (blue-shifted) wavelength effectively increased the number of localized single molecules of these fluorophores. *For Alexa 647, Atto 655, and Alexa 660, addition of 3–5 mM MEA in the buffer improved the blinking with no significant impairment of the performance of VdcV. ^$^For Alexa 555 and Alexa 568 a protocol of dual color SMLM imaging does not necessitate correction of the chromatic shift. We reported on the use of DAPI and Hoechst dyes (photoproducts) in SMLM previously [3].
