# Supplementary material for: Quantitative super-resolution localization microscopy of DNA in situ using Vybrant® DyeCycle™ Violet fluorescent probe
Source: Data Brief. 2016 Jan 29;7:157–71. doi: 10.1016/j.dib.2016.01.041 (PMC4802433; doi:10.1016/j.dib.2016.01.041)
Supplement: Supplementary file 2 — Supplementary material [file mmc2.pdf]

SUPPLEMENTARY MATERIALS / DATA IN BRIEF

Quantification in Localization microscopy of DNA in situ using Vybrant® DyeCycle™ Violet Fluorescent Probe: A new approach to study nuclear nanostructure at improved resolution

Dominika Żurek-Biesiada[a] , Aleksander T. Szczurek[b], Kirti Prakash[b,c], Gerrit Best[d], Giriram K. Mohana[b], Hyun-Keun Lee[b,e], Jean-Yves Roignant[b], Jurek W. Dobrucki[a,+], Christoph Cremer[b,c,d,e+], Udo Birk[b,d,f]

Affiliations:

[a]Laboratory of Cell Biophysics, Faculty of Biochemistry, Biophysics and Biotechnology, Jagiellonian University, Gronostajowa 7, 30-387, Kraków, Poland,

[b]Institute of Molecular Biology (IMB), Ackermannweg 4, 55128, Mainz, Germany,

[c]Institute for Pharmacy and Molecular Biotechnology (IPMB), University of Heidelberg, Im Neuenheimer Feld 364, D-69120, Heidelberg, Germany,

[d]Kirchhoff Institute for Physics, University of Heidelberg, Heidelberg, Germany.

[e]Department of Physics, University of Mainz (JGU), Staudingerweg 7, 55128, Mainz, Germany.

[+]***corresponding authors:*** c.cremer@imb-mainz.de; +49 (0) 6131-39- 21518; jerzy.dobrucki@uj.edu.pl; +48 12 664-63-82

Abstract

Single Molecule Localization Microscopy (SMLM) is a recently emerged optical imaging method that was shown to achieve a resolution in the order of tens of nanometers in intact cells. In this data article, in support of research work entitled "Localization microscopy of DNA in situ using Vybrant® DyeCycle™ Violet Fluorescent Probe: a new approach to study nuclear nanostructure at single molecule resolution ", we provide quantitative information on the influence of the chemical environment on the behavior of the dye, discuss the variability in the DNA-associated signal and demonstrate direct proof of enhanced structural resolution. Furthermore, we compare different visualization approaches. Finally, we describe various opportunities of multicolor DNA/SMLM imaging in eukaryotic cell nuclei.

Specifications Table

Subject area	*Biology*
More specific subject area	*Super-resolution microscopy of cell nuclei*
Type of data	*Tables, Figures*
How data was acquired	*Super-resolution localization microscopy data based on blinking of Vybrant DyeCycle Violet dye with an affinity to nuclear DNA; 2D data constitute an optical slice (<500 nm thickness) through cell nuclei*
Data format	*Analyzed SMLM list of positions and reconstructions*
Experimental factors	*VERO-B4 cell nuclei stained with a dye at a variety of concentrations and chemical environments*
Experimental features	*Blinking of the green-emitting form of the dye induced by single 491 nm laser of high intensity*
Data source location	*Mainz, Germany, Krakow, Poland*
Data accessibility	*The data are with this article*

Value of the data

- Single molecule localization microscopy of DNA based on blinking of the photoproduct is possible with single wavelength excitation yielding improved structural resolution
- A number of other fluorescent probes perform well in the imaging conditions used for this DNA dye, thus enabling multicolor single molecule localization microscopy of DNA and, for instance, immunofluorescently labelled targets
- Optical investigation of nuclear nanostructures necessitates new DNA imaging methods providing enhanced resolution and efficiency as compared to the presently available techniques

Experimental Design, Materials and Methods

Single Molecule Localization Microscopy (SMLM) is an imaging method based on blinking of fluorophores, namely, inducing transient emission and following detection of up to 10^4 photons from an individual (optically isolated) fluorophore. The position of such a single point emitter can be extracted with nanometer precision by fitting the diffraction limited signal detected on the CCD camera. In the following report we present supportive data for our work on SMLM imaging of photoconvertible DNA dyes. Here we present quantitative evaluation of the properties of Vybrant® DyeCycle™ Violet (VdcV) and its green-emitting photoproduct, when the dye is attached to DNA. Optical isolation is achieved by conversion of a small fraction of VdcV molecules to the green emitting form, and detection of the fluorescent signal in the green channel only.

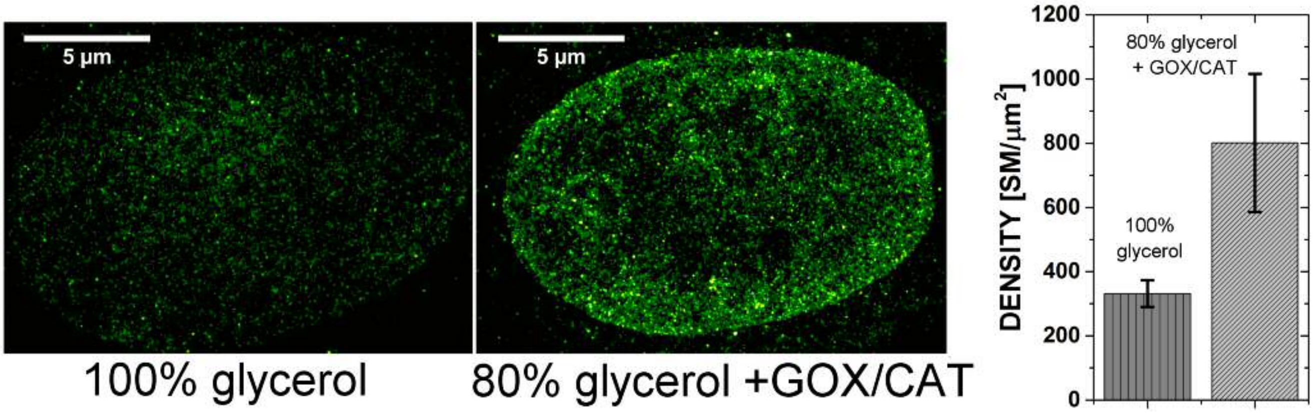

Influence of the embedding buffer on blinking of the green-emitting form of Vybrant® DyeCycle™ Violet

Supplementary Figure S1. The influence of the enzymatic oxygen scavenging system (abbreviated as GOX/CAT), diluted in PBS and added to glycerol, on SMLM image quality. Under the conditions used in Fig. 1, glycerol alone provided an average density of about 300 single molecule (SM) signals per μm^2, or 17.3 x 17.3 SM/μm^2, i.e. one signal per 1000/17 nm = 59 nm, corresponding to an average estimated structural resolution of ~ 2 x 59 nm. However, by adding the specifically designed enzymatic oxygen scavenging medium, the average density of SM signals was increased several times, resulting in an SMLM image with further enhanced structural resolution (based on the same number of image frames). For a quantitative comparison we used only VdcV at 10 nM, i.e. at a concentration where saturation of the detector does not occur at the beginning of the illumination procedure. This protocol made it possible to run a measurement without prior bleaching. Using an excitation wavelength of λ_{exc} = 491 nm (illumination intensity 0.3 kW/cm^2) and fluorescence emission registration in the range of 585 - 675 nm, 20,000 frames were collected. Signal densities obtained in the images of the cell nuclei: 207 SM/μm^2 in 100% glycerol (n=7) and 679 SM/μm^2 in the imaging buffer (n=9; n= number of cells analyzed). 207 SM/μm^2 (= 14.4 x 14.4 SM μm^2) corresponds to a next neighbor distance of one signal per 1000/14.4 nm = 70 nm, resulting in an average estimated structural resolution of ~ 2 x 70 nm; 679 SM/μm^2 (=26 x 26 SMμm^2) corresponds to a next neighbor distance of one signal per 1000/26 nm = 38 nm, resulting in an average estimated structural resolution of ~ 2 x 38 nm. Note that the optical (two point) resolution depends on the precision of localization , whereas the structural resolution depends also on the density of signals, thus it may be different for different SMLM images, even if the localization precision remains the same.

In search for the optimal imaging conditions for SMLM, potentially in combination with Structured Illumination Microscopy (SIM) (Rossberger et al., 2013), a number of imaging buffers, with various components were tested, including: PBS, glycerol, mercaptoethylamine (MEA), glucose oxidase in combination with catalase and glucose, ascorbic acid and Prolong Gold®. All of these factors may influence the number of fluorescent cycles (i.e. photons emitted by a single molecule) during an 'ON'-state. Note that the number of emitted photons per fluorescent molecule (N) directly influences localization precision, the latter being proportional to $N^{-1/2}$. The observed large differences in the number of signals detected from the green-emitting form of Vybrant® DyeCycle™ Violet depend on

the concentration of the oxygen scavenging system, i.e. on the oxygen content. As reported (Bernaś et al., 2004), oxygen is the primary source of irreversible damage inflicted upon the fluorophore, especially upon high intensity illumination; such permanent photobleaching results in a deterioration of the image quality (localization precision, density of molecule signals).

All of the buffers mentioned above were tested in combination with various concentrations of VdcV (Supplementary Table 1). The most prominent and efficient blinking of the green-emitting form of VdcV was observed when an enzymatic oxygen scavenging system, at a relatively low concentration, dissolved in glycerol, was used (Supplementary Fig. S1).

Supplementary Table 1

no.	imaging buffer	blinking	bleaching	comment
**1**	**aqueous PBS**	+	- - - -	Single molecule fluorescent bursts have low SNR and are sparse immediately after applying illumination.
**2**	**glycerol**	++	- - -	Strong signals at the beginning, relatively fast bleaching, comparison in Supplementary Fig. S1.
**3**	**80% glycerol + 20% PBS (comprising finally 0.5 mg/ml glucose oxidase, 0.04 mg/ml catalase, 0.1 g/ml glucose)**	++++	-	Buffer used previously for bisbenzimide dyes (Szczurek et al., 2014). For high concentrations of the dye, the CCD detector is easily saturated and the signal bleaches slowly; for low concentrations of the dye the detector is less likely to be saturated, but the signal decreases swiftly due to rapid photobleaching. A successful use of the buffer containing glucose oxidase - catalase system in PBS for SMLM of some standard fluorophores was reported (Dempsey et al., 2011), see Supplementary Fig. S1.
**4**	**80% glycerol + 20% PBS (comprising finally 0.5 mg/ml glucose oxidase, 0.04 mg/ml catalase, 0.1 g/ml glucose) + MEA**	+++	-	Addition of 10 mM MEA to either PBS or glucose oxidase - catalase system was proven to induce blinking in most of the fluorophores covering the entire visible spectrum of fluorescence emission (Dempsey et al., 2011) indicating its potential in multicolor experiments. An addition of up to 5 mM MEA (cysteamine) to our optimized buffer did not hamper blinking of VdcV significantly. Higher concentrations reduced blinking strongly.
**5**	**glycerol + 10 % PBS**	++	- - - -	A high number of signals with moderate SNR was detected at the beginning of the measurement, followed by a rapid decline of blinking events.
**6**	**Prolong Gold®**	+	- - -	Very low SNR due to high background and low intensity of fluorescent bursts. This standard embedding medium was already reported several times to perform best for AlexaFluor 488 and 594 (Kaufmann et al., 2012) or standard fluorescent proteins (Lemmer et al., 2008; Kaufmann et al., 2009).

7	**90% glycerol + 10% 10 mM ascorbic acid in PBS**	+++	- - -	High SNR of blinking molecules, fast bleaching disabling further acquisitions after 1 - 2 minutes. A similar buffer was proven to be optimal for imaging of PicoGreen-stained DNA (Benke and Manley, 2012) and various dyes with specific affinity to several cellular organelles (Carlini and Manley, 2013).
8	**10 mM ascorbic acid in PBS**	+	- - -	Low SNR of blinking molecules, fast bleaching
9	**95% glycerol + 5% PBS (comprising finally 0.25 mg/ml glucose oxidase, 0.02 mg/ml catalase, 0.05 g/ml glucose in PBS)**	++++	- -	A low concentration of enzymes performing oxygen scavenging was insufficient to prevent bleaching. This precluded long experiments, i.e. less single molecule fluorescent bursts can be recognized in multiframe acquisitions.
10	**PBS + 100 mM MEA**	+	- - - -	Hardly any blinking of VdcV was observed. This switching buffer used for AlexaFluor dyes was originally reported by (Heilemann et al., 2008), later shown to work for a broad spectrum of synthetic dyes (Dempsey et al., 2011).

Supplementary Table 1. Qualitative description of various imaging buffers influencing the number of detected molecules of the green-emitting form of VdcV, detected in SMLM imaging of DNA in Vero-B4 nuclei. SNR - signal-to-noise ratio; '+' observed blinking rate ('++++' is best for high optical and structural resolution SMLM of nuclear DNA); '-' extent of bleaching during experiments. None of the buffers listed above prevented photoconversion of VdcV.

Conversion of Vybrant Violet to its green-emitting form

The localization microscopy based on VdcV reported here shares some similarities with the results reported for SMLM imaging of Hoechst 33258, Hoechst 33342 and DAPI (Szczurek et al., 2014). In our recent publications (Żurek-Biesiada et al., 2013; Żurek-Biesiada et al., 2014) we described the properties of these dyes using mass spectrometry and found a significant dependence of the abundances of the protonated forms of Hoechst 33258 on pH and the presence of hydrogen peroxide. A similar blinking behavior of Hoechst dyes and VdcV, and the fact that we used the same imaging buffers to induce blinking, suggest that the photophysical mechanism underlying the conversion of the blue-emitting to the green-emitting form of VdcV may be protonation, as it was demonstrated for bisbenzimide dyes (Żurek-Biesiada et al., 2013). Further studies are required to understand the photophysics of VdcV on the single molecule level in the context of the chemical environment.

From a general methodological point of view it may be noted that the SMLM method used here largely simplifies the approach to super-resolution microscopy, as it requires only monochromatic illumination for both photo-switching and fluorescence read-out; standard (or slightly modified)

sample preparation; and a single type of organic fluorophores. Previously, it has been successfully applied in other cases, such as Alexa and Atto dyes and green fluorescent proteins (Cremer et al., 2011; Reymann et al., 2008; Lemmer et al., 2008). In this report, it is shown that a very similar approach can be effectively used to achieve SMLM imaging of nuclear and chromosomal DNA distribution directly stained with VdcV, as the single laser wavelength not only induces blinking, but it may also be used to induce photoconversion, resulting in a red-shift of the emission wavelength.

Single molecule fluorescent bursts - raw data

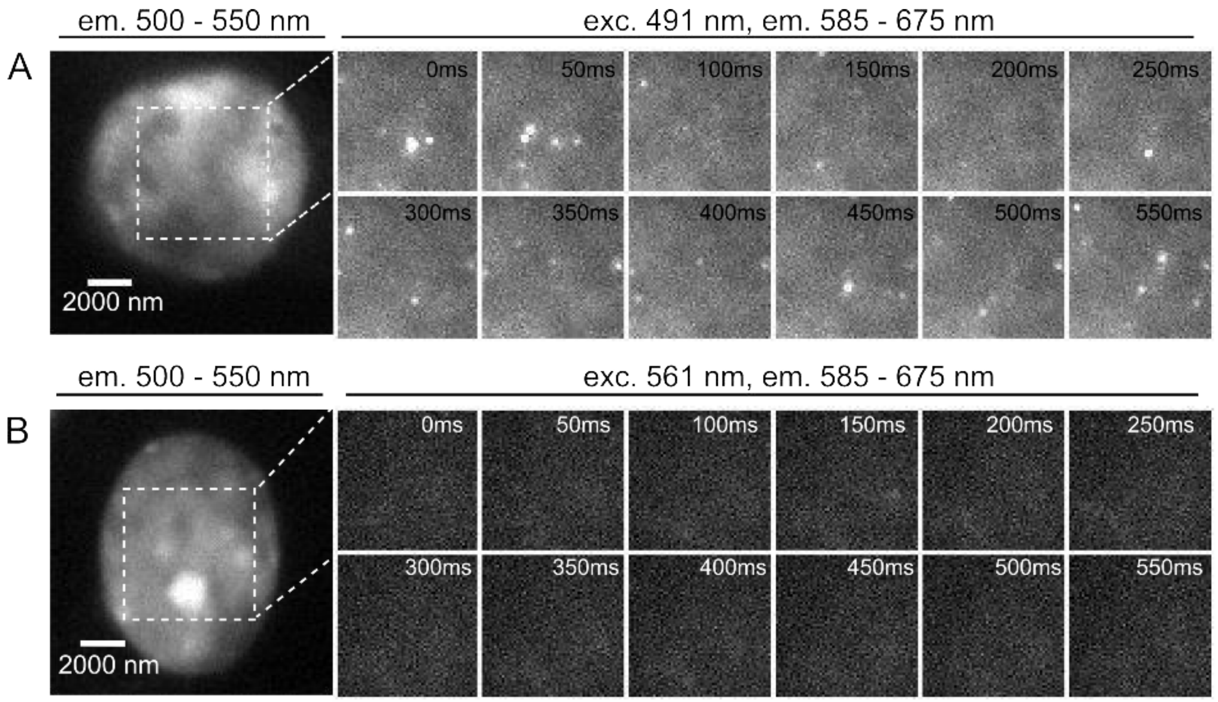

Supplementary Figure S2. Single molecule fluorescent bursts in Vybrant dye cycle Violet stained cells detected in the green-yellow emission range (585 - 675 nm) using high intensity single wavelength excitation (0.525 kW/cm^2, λ_{exc} = 491 nm) after several minutes of illumination (A). Molecules of the green-emitting form of VdcV (occurring naturally under standard conditions) were reversibly bleached and reappeared stochastically in the green-yellow detection channel. Concentration of VdcV was 500 nM. Note that the relative time values given may actually slightly differ from the real ones since the read-out time of the camera is not taken into account. (B) Single molecule localization acquisition in the same detection channel using high excitation intensity 561 nm illumination (appropriate for blinking of Alexa555 or Alexa568), in the same detection channel as the second reporter molecule, distinguished on the basis of distinct excitation spectra. As can be inferred from the raw data images, blinking induced by 561 nm excitation is negligible in the absence of Alexa reporter molecules. However, we noted that some blinking did appear and was mostly associated with the cytoplasm.

Measurement of resolution in VdcV/SMLM data

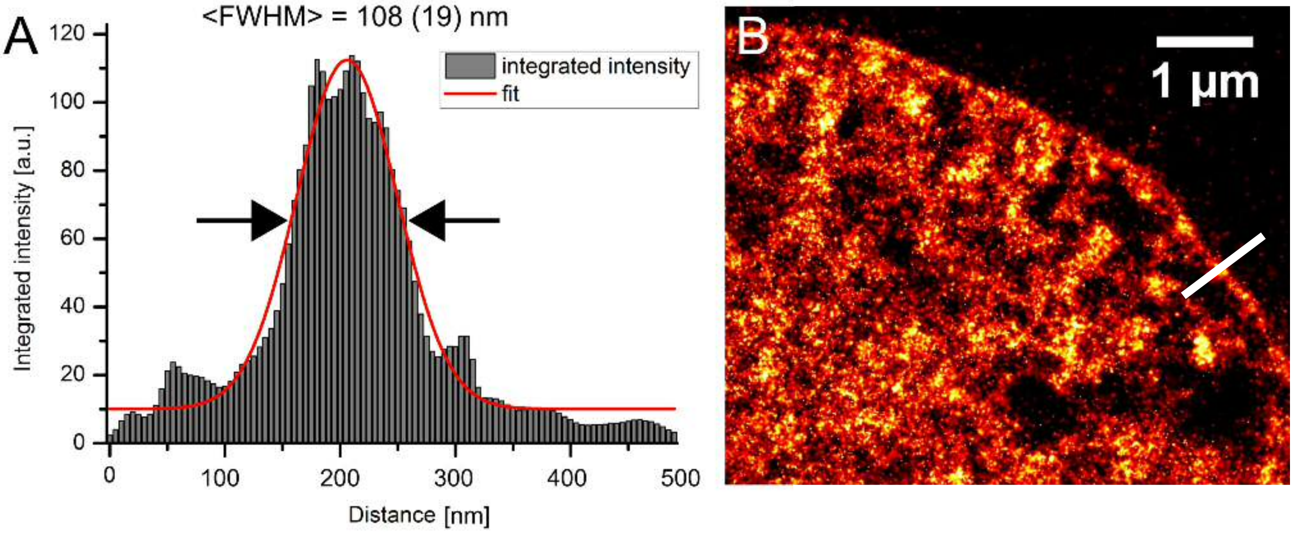

Supplementary Figure S3. Sub-diffraction measurement of heterochromatin aligned to the nuclear envelope in HL-1 cells. The thickness of this chromatin structure is known to be low, and the structure highly compacted. It has been measured after fitting a Gaussian function and equals 108(±19) nm (n=5). Integration has been performed in 100 nm x 500 nm rectangle regions.

A - An example of an intensity profile across a heterochromatin region (as highlighted in B by the white line) at the nuclear membrane.

B – Nucleus in which the measurement has been performed.

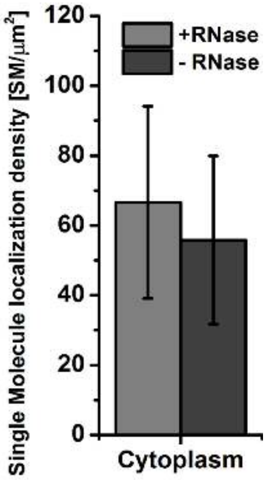

Supplementary Figure S4. Single Molecule Localization data acquired in the cytoplasm. 1h RNase treatment at 37°C does not influence the density of single molecule localizations in the cytoplasm indicating that contribution to the total signal arising from Vybrant Violet bound to RNA was negligible (n=3).

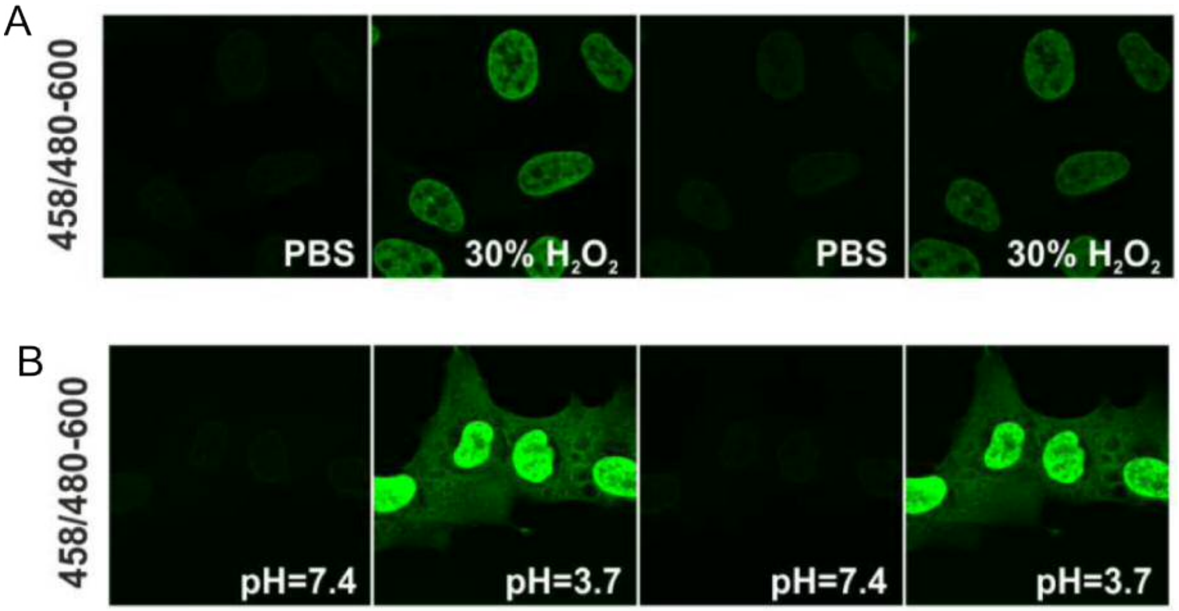

Supplementary Figure S5. Vybrant Dye Cycle Violet fluorescence properties are altered by low pH or a high concentration of hydrogen peroxide. MSU 1.1 cells were stained with Vybrant DyeCycle Violet at 1 µM, green emission of VdcV excited by 458 nm was recorded in the 480 - 600 nm band and chemical environment was exchanged as stated in the image panels. Images presented here were acquired using constant excitation intensity, and were acquired sequentially within minutes. This behaviour of Vybrant Dye Cycle Violet strongly resembles the behaviour of DAPI and Hoechst dyes, as we previously reported (Żurek-Biesiada et al., 2013). Note that the changes reported here are reversible. The experimental procedures were reported previously (Żurek-Biesiada et al., 2013). The binding mode of VdcV is not disclosed, however properties resemble the properties of minor-groove binders just as Hoechst.

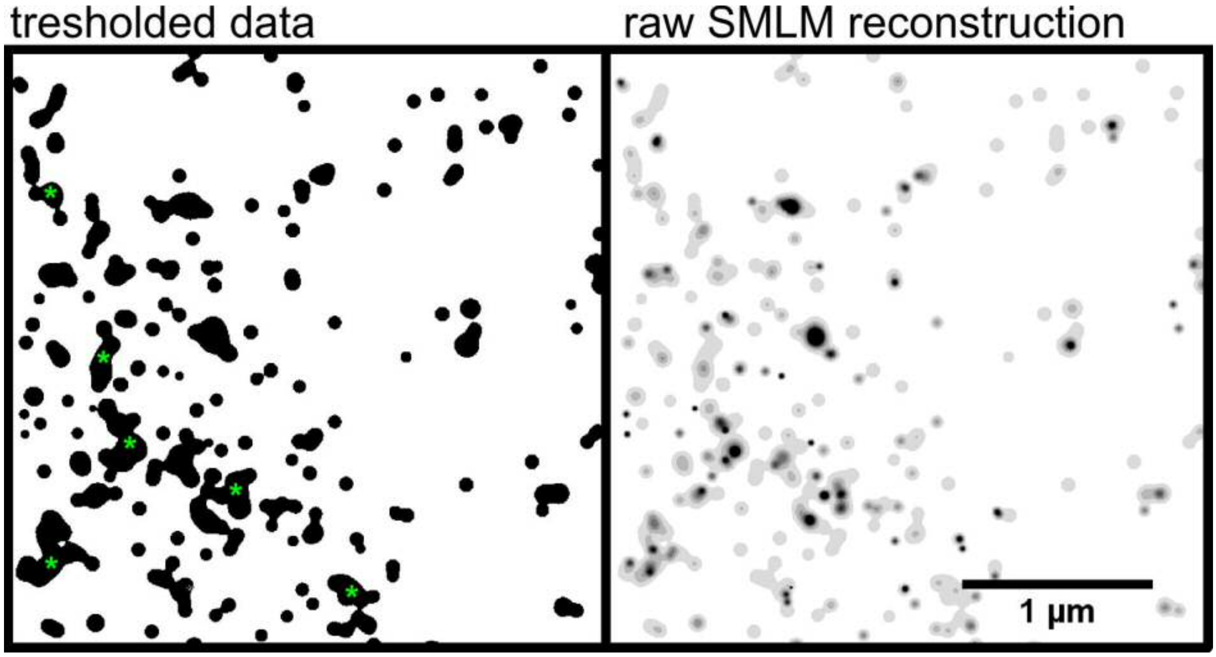

Supplementary Figure S6. In order to provide an estimate of the number of re-appearing single molecule events in our SMLM images the cytoplasm containing mitochondrial DNA and RNA was analyzed. First the SMLM image of VdcV-stained cells was acquired (30,000 frames, 50 ms exposure time). Then the reconstructed raw SMLM image (example in right) was tresholded in order to include all pixel values >0 to the binary mask (left). Next the number of objects in the binary mask was calculated and total number of single molecule localizations was attributed to the number of binary objects. The number of objects in the binary mask

amounted to 13,8(1,2)/µm^2, which is more than the number of theoretical Airy discs fitting in an area of a square micron (roughly 3). Commonly, clearly overlapping single molecule signals are present in such analysis (indicated with green asterisks) and they will contribute to overestimating the number of single molecule localizations per single molecule of VdcV.

Dependence of the number of single molecule bursts, detected in a single excitation experiment, on the concentration of VdcV and the length of image acquisition

We tested various concentrations of VdcV, ranging from 10 to 1000 nM. Using a 20,000 frame protocol and a 491 nm excitation, we detected very high numbers of fluorescent bursts that increased with the increasing concentration of the dye. Differences in the range of 300 nM - 1000 nM VdcV were not significant, perhaps due to different bleaching times that were applied prior to the measurements. Examples of SMLM images acquired using different concentrations of VdcV are shown in Supplementary Fig. S7.

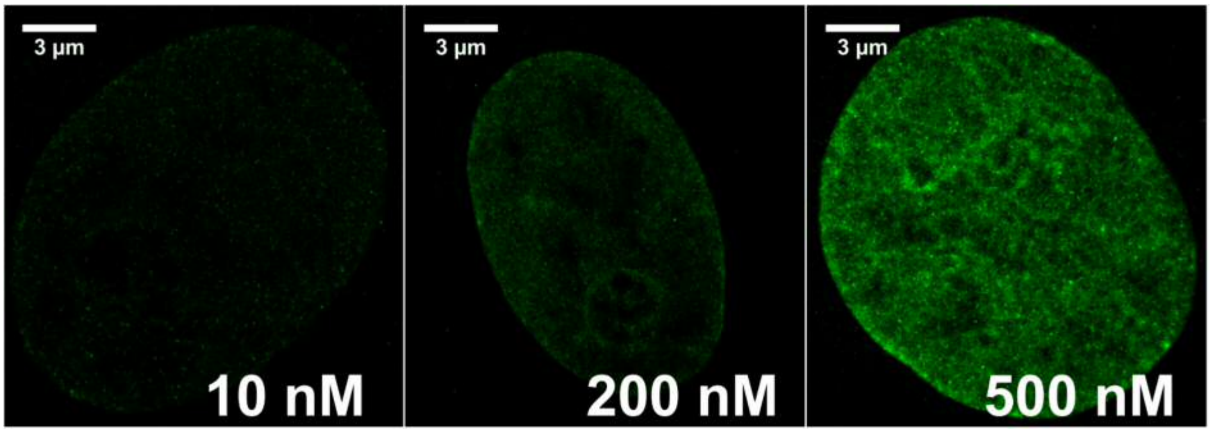

Supplementary Figure S7. Different concentrations of VdcV bound to nuclear DNA influence the signal density in the reconstructed images. Images of Vero-B4 nuclei were acquired using SMLM with high intensity 491 nm illumination (0.525 kW/cm^2) for three concentrations of VdcV (10 nM, 200 nM, 500 nM). We observed the following average signal densities in the detection channel (λ_{em}: 585 - 675 nm): 944 single molecules/µm^2 (10 nM), 1735 single molecules/µm^2 (200 nM) and 4818 single molecules/µm^2 (500 nM). 20,000 frames were recorded in each measurement.

Subsequently, we investigated how the total acquisition time i.e., the number of acquired frames, influenced the number of detected single fluorescent bursts. First, the cells were subjected to a pre-bleaching period, during which the pool of molecules emitting in the green-yellow range was drained. After this period, we found that the total number of single fluorescent bursts detected depends almost linearly on the number of frames acquired (Fig. S8), irrespective of the local signal density in the nuclear sample. The exponential decay, i.e. the irreversible photobleaching amounts to less than 6% for the 20,000 frames acquired (least squares regression). Figure 5 demonstrates that it

is possible to acquire a satisfactory SMLM image within 5,000 - 10,000 frames (total acquisition time approximately 8 minutes) yielding structural details that cannot be obtained by standard widefield microscopy.

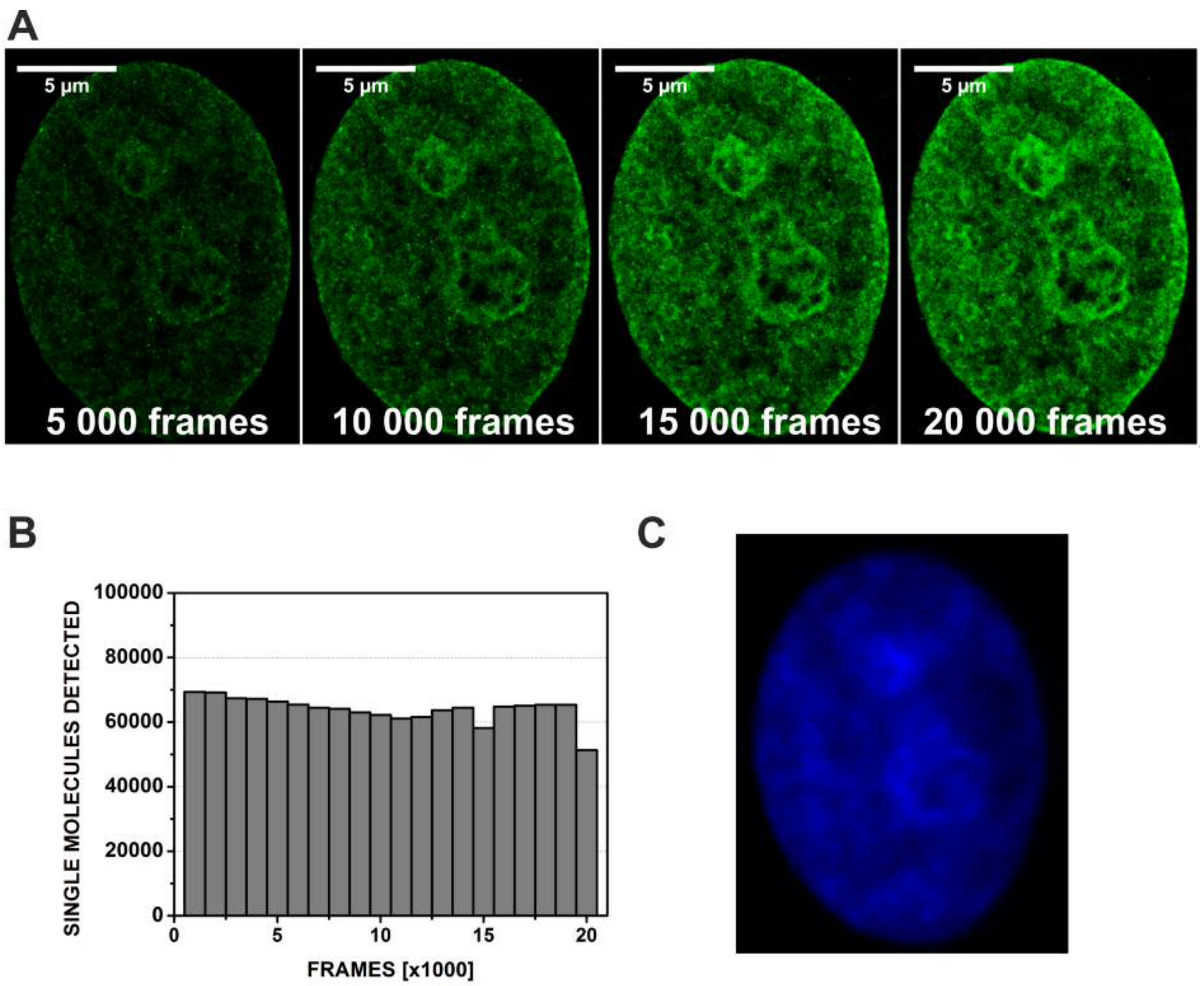

Supplementary Figure S8. Dependence of the SMLM image quality on the duration of the image acquisition.

A - Reconstructed images of a Vero-B4 cell nucleus stained with 500 nM Vybrant® DyeCycle™ Violet. The images show that the total number of single molecule fluorescent bursts detected depends strongly on the total number of the image frames, i.e. on the length of the image acquisition. Average signal densities for the images: 1496 single molecules/µm^2 (5,000 frames); 2901 single molecules/µm^2 (10,000 frames), 4285 single molecules/µm^2 (15,000 frames), 5716 single molecules/µm^2 (20, 000 frames). SM signals per frame (in the following denoted by α): 5,000 frames, α = 1496/5,000 = 0.299 SM/frame; 10,000 frames, α = 0.290; 15,000 frames, α = 0.286; 20,000 frames, α = 0.286; λ_{exc} = 491 nm (0.525 kW/cm^2), λ_{em}: 585 - 675 nm.

B – Dependence of the number of the single molecule signals detected per 1000 acquired frames on acquisition time. A slight permanent bleaching (exponential decay) is observed.

C – A widefield image of fluorescence of VdcV in the same Vero-B4 cell nucleus; λ_{exc}= 405 nm, λ_{em}= 440 - 490 nm.

Localization precision and Photon Count in SMLM measurements of DNA-bound VdcV in the cell nucleus

Figure 6 depicts statistics of individual signals extracted after acquiring photoconverted DNA-bound VdcV as visualized in Figure 2.

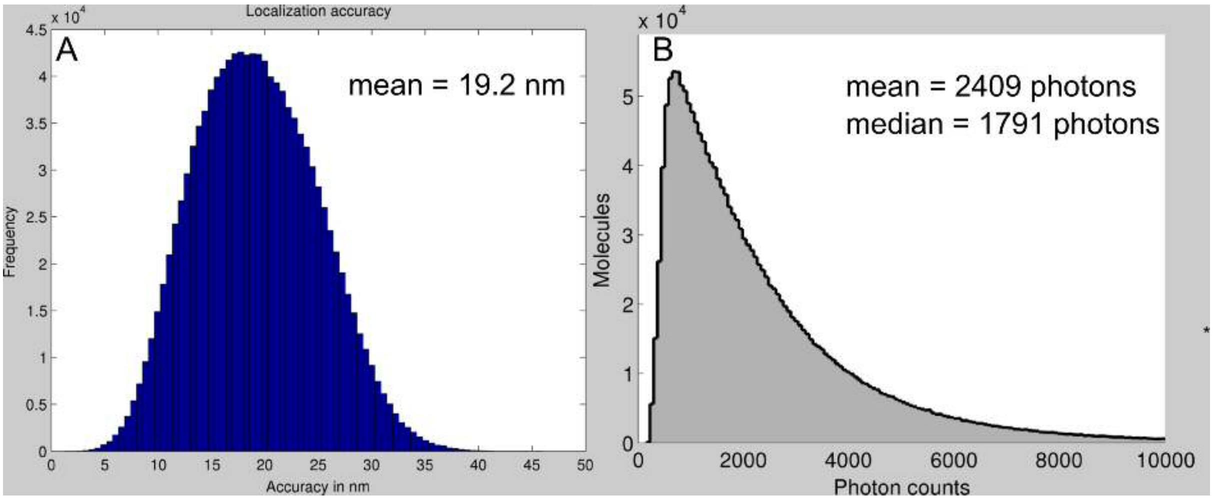

Supplementary Figure S9. Distribution of the values of localization precision (A) and photon counts (B) obtained for measurements of DNA-bound VdcV in the cell nucleus.

A - Histogram of individual localization precision. The ordinate gives the frequency (number) of individual molecule signals in the cell nucleus evaluated with a given localization precision (abscissa).

B - Histogram of photon counts: the ordinate gives the number of molecules with a detected fluorescence photon count (abscissa).

Visualization of SMLM measurements of DNA-bound VdcV in the cell nucleus

In Supplementary Figure S10, different visual representations of SMLM data, i.e. of signals extracted after acquiring photoconverted DNA-bound VdcV are shown, for the dataset which was visualized in Figure 2.

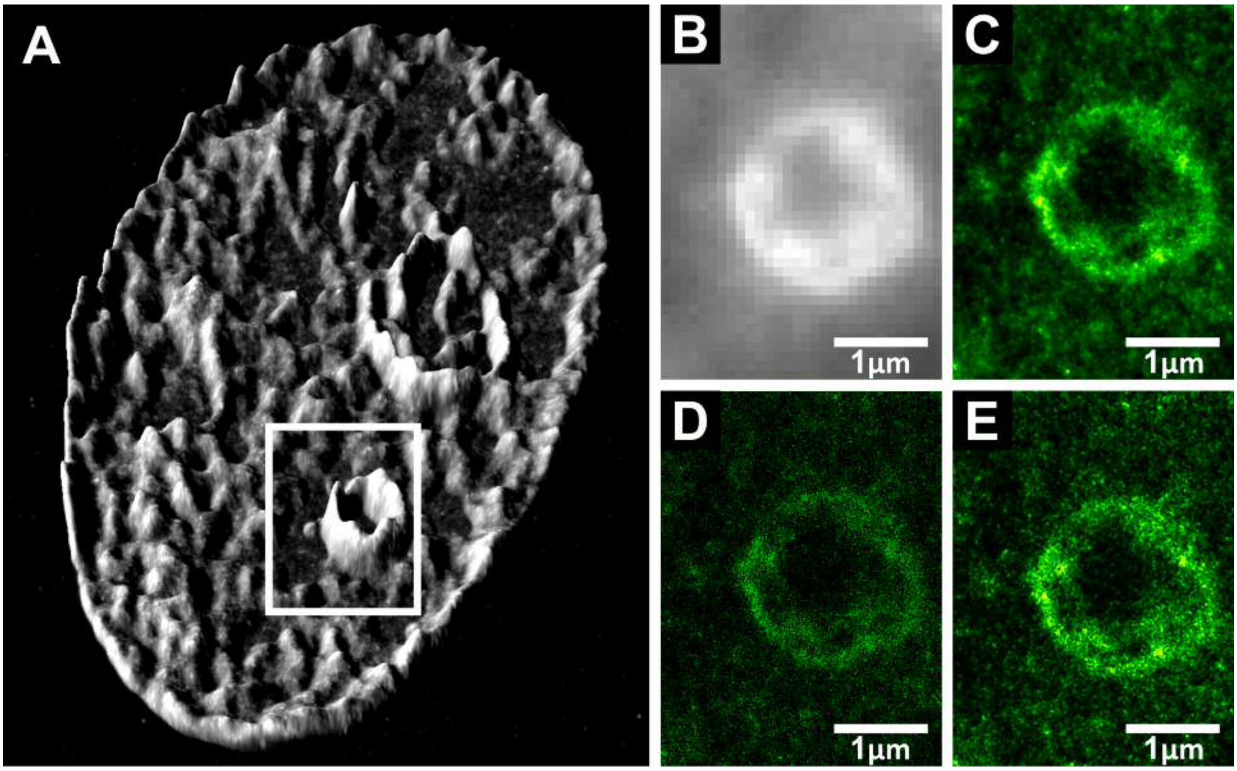

Supplementary Figure S10. Different methods of presenting high-resolution localization microscopy data and a comparison with a widefield image.

A - perinucleolar chromatin stained with VdcV and imaged with SMLM. Height (the 3rd dimension) designates the density of the signal (the number of single molecules/µm^2) of the reconstructed image in Figure 2. The SMLM image was produced after smoothing.

B - a widefield image of the region marked in A.

C - data points blurred with the respective localization precision.

D - point representation of single molecule positions.

E - triangulation originally described by (Baddeley et al., 2010) run over the single molecule data set.

λ_{exc} = 491 nm, 0.525 kW/cm^2, λ_{em}: 585 - 675 nm.

Profiles extracted from reconstructions of DNA-bound VdcV in the cell nucleus

In Supplementary Figure S11 and S12 the same data set was used as visualized in Figure 2.

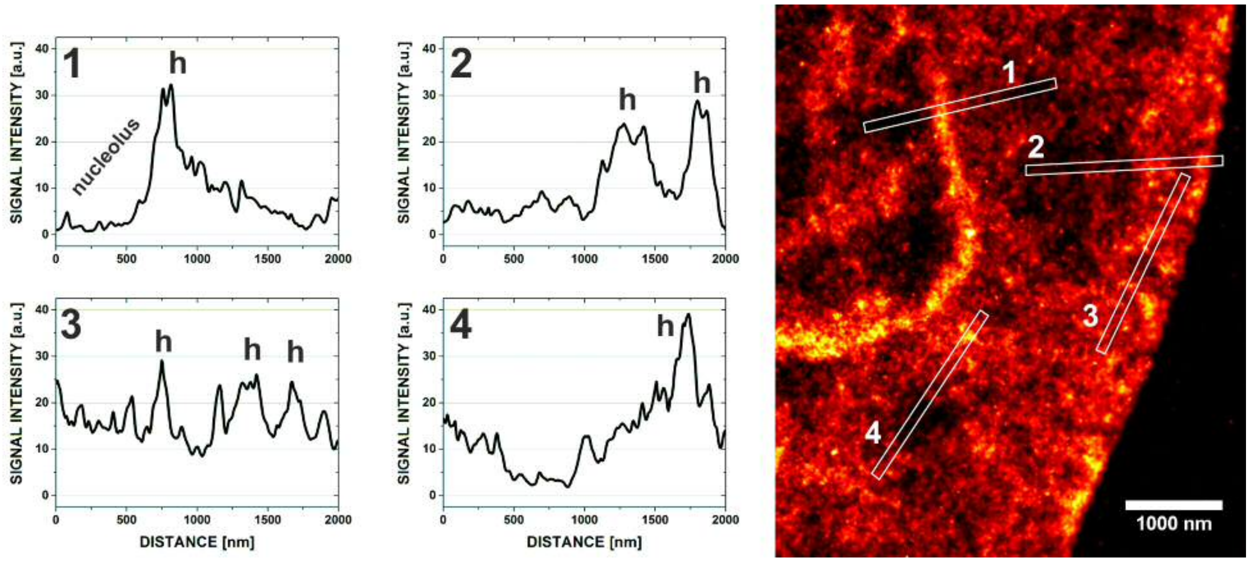

Supplementary Figure S11. Plot profiles (left, center) indicating signal density differences occurring on the nanoscale in the reconstructed image of a Vero-B4 cell nucleus (right) stained with 500 nM Vybrant® DyeCycle Violet. The regions most likely representing heterochromatin are marked in the plot profiles with 'h'. Plot profiles were obtained by integrating the signals over rectangular regions 2 µm long and 100 nm wide. The graphs show the signal density profile in: perinucleolar heterochromatin (1); euchromatin and heterochromatin across the nuclear periphery (2); heterochromatin and euchromatin in the vicinity to the nuclear envelope (3); the lowest DNA density region in an inner region of the nucleus (4).

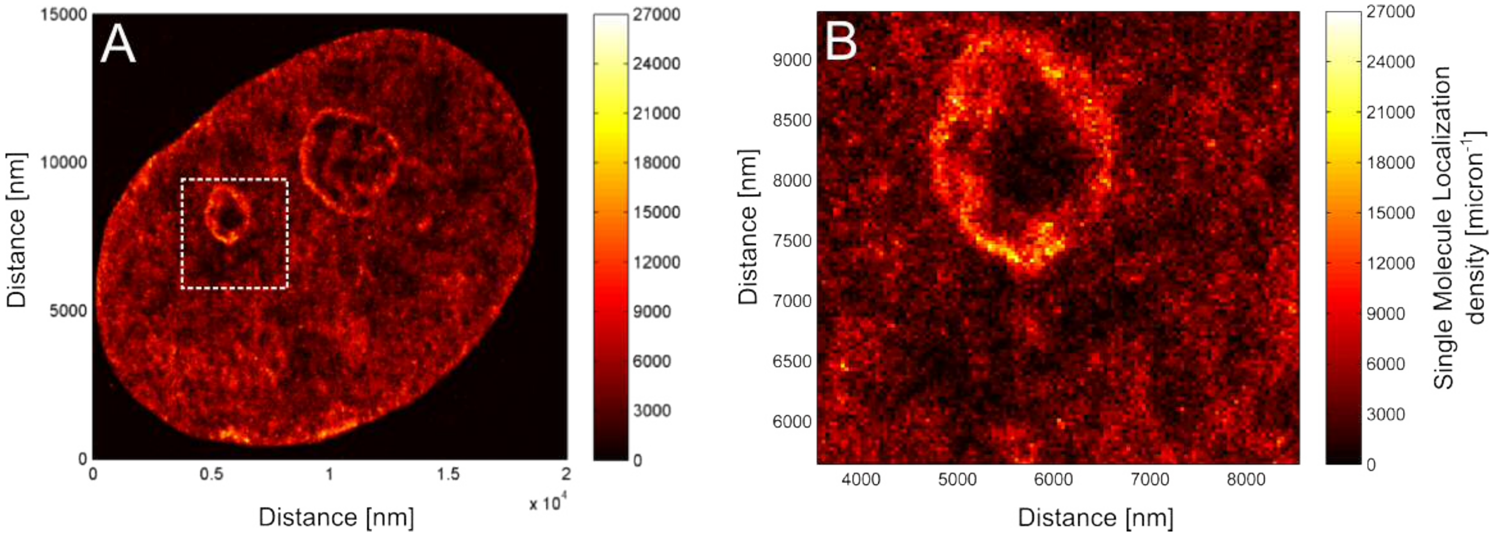

Supplementary Figure S12. DNA density is visualized after binning the single molecule localizations into a 40nm x 40nm grid of pixels. The density presented here is given as the number of localizations per square micron. As can be seen from the color-coded scale the density of localizations within the image varies by a factor ~20 indicating the existence of DNA-poor areas in the cell nucleus, likely attributable to the Interchromatin Compartments (IC)(Cremer et al., 2015). Note the low signal of Vybrant Violet inside the nucleoli.

Study of the influence of photoconverting 405 nm illumination on VdcV fluorescence bursts

In Supplementary Figure S13, the effects of additional 405 nm illumination on the performance of the SMLM measurements are shown. The analysis was performed using the same dataset as visualized in Figure 3.

In order to investigate the influence of low intensity 405 nm illumination on SMLM measurements, namely on the appearance of single molecule fluorescent bursts, we stained Vero-B4 cells with VdcV at a very low concentration, i.e. 50 nM, allowing for an immediate beginning of the measurement rather than performing the step of pre-bleaching of the excessive amount of the fluorescence signal prior to the measurement. Single molecules of the green-emitting form of VdcV were easily isolated from the relatively low background immediately after applying 1.2 kW/cm^2 of 491 nm excitation. However, when we applied a linearly decaying ramp of 405 nm laser illumination with a very low initial illumination intensity of 2 W/cm^2 and a duration (decay time) of approx. 1 minute, a significantly higher number of single molecule fluorescence bursts was detected, with a corresponding higher number of the simultaneously fluorescing molecules from the same focal plane

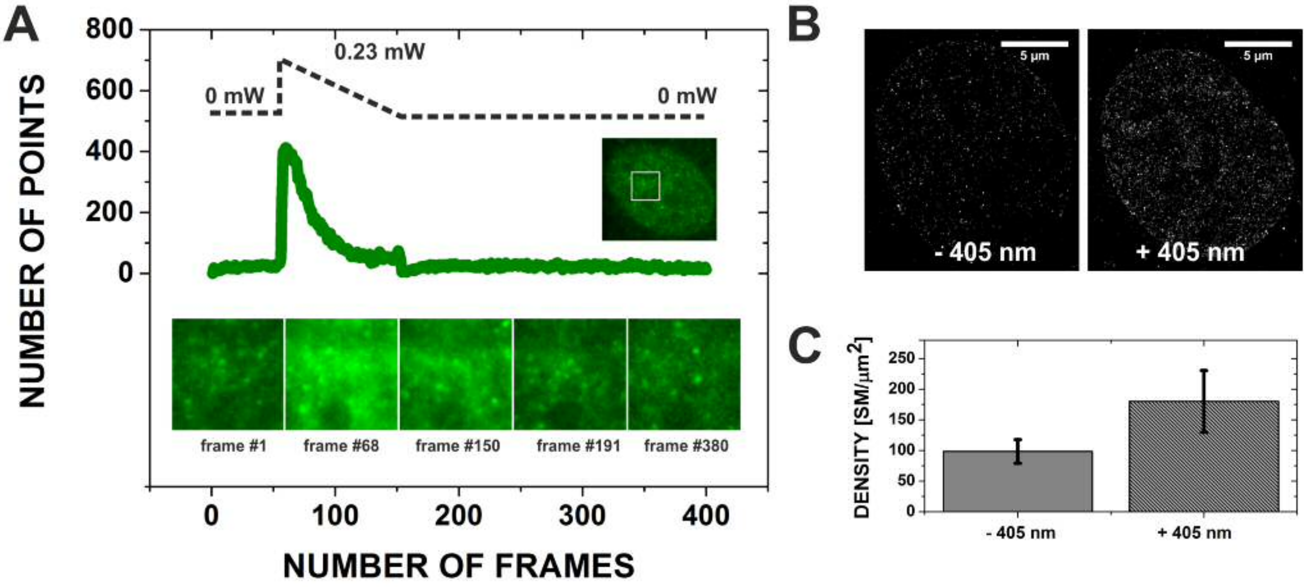

(see Supplementary Fig. S13).

Supplementary Figure S13. The influence of 405 nm light illumination on SMLM measurements of the green-emitting form of VdcV (150 nM).

A - When 405 nm was applied to the sample, which was already being illuminated with 491 nm excitation, an immediate increase in the number of points detected per frame was detected (frames: 50 - 200). $\lambda_{exc.}$ = 491 nm, 0.525 kW/cm^2, λ_{em}: 585 - 675 nm.

B - Examples of SMLM measurements of cells stained with 50 nM VdcV (low concentration) in the presence or absence of 405 nm light (110 µW). When this concentration of VdcV was used, no pre-bleaching was required. Therefore, it was feasible to assess the influence of the photoconverting 405 nm illumination on the number of molecule signals detected. 5,000 frames were collected. λ_{exc} = 491

nm, 0.4 kW/cm^2, λ_{em}: 585 - 675 nm.

C - A graph presenting the density of single molecule fluorescent bursts detected under both conditions (abbreviated as SM). (-) Without 405 nm excitation; (+) with additional 405 excitation. Each bar presents an average (and standard deviation) from five experiments.

Resolution of the SMLM System, when applied to 1-dimensional samples

Most published SMLM implementations have been optimized for analysis of 1D or 2D structures. Measurements of such structures have been reported typically with a precision in the order of 10 nm. In our approach to visualizing chromatin in 3D intact cell nuclei, we have reported a resolution equivalent in the order of 100 nm, in spite of the microscope setup being able to produce SMLM data with much higher resolution. In Supplementary Figure S14, the results of SMLM measurements of the diameter of fluorescently labeled microtubules are shown, indicating that much higher resolution (37 nm) is achievable, under conducive conditions, with the instrument used in our study of chromatin.

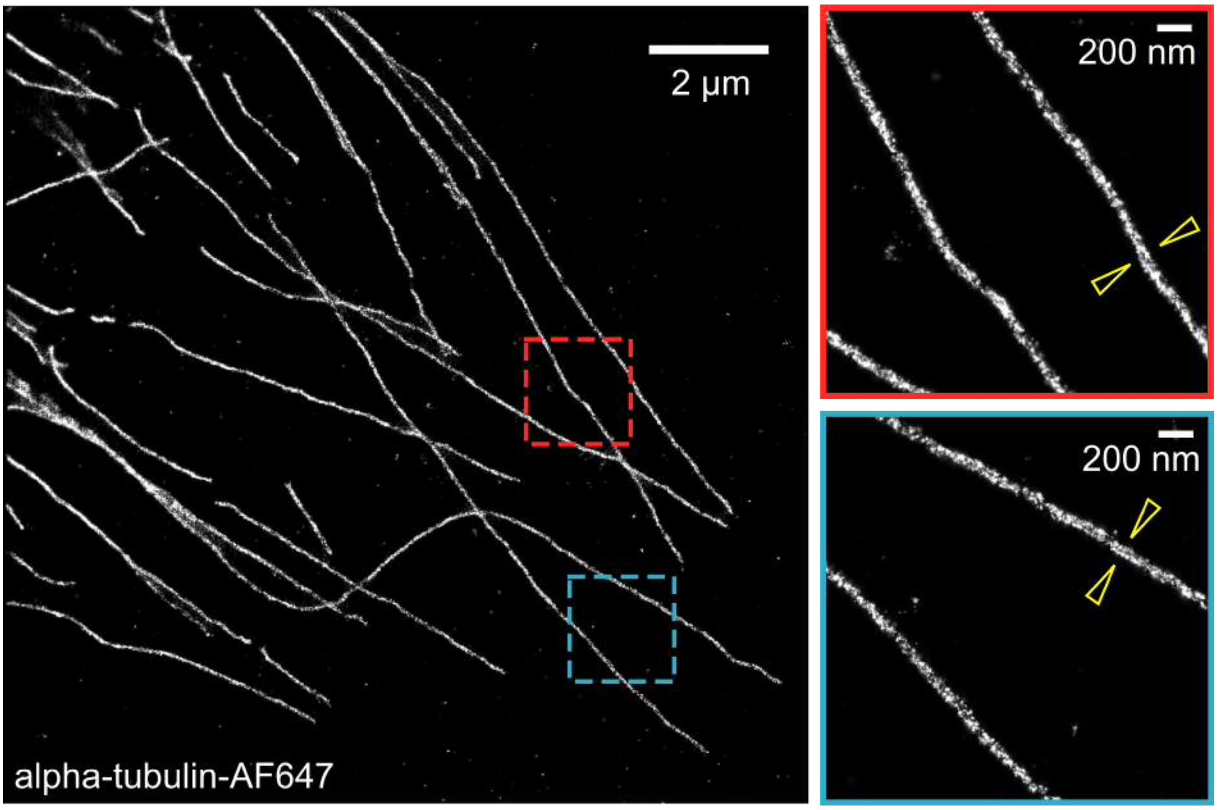

Supplementary Figure S14. SMLM microscope calibration with a standard specimen of immunofluorescently labelled α-tubulin. Blinking of Alexa 647 (which was conjugated to the secondary antibody) was induced in the presence of primary thiol-containing imaging buffer devoid of oxygen. Reconstruction was performed with the software, which was used also for analysis of DNA/SMLM data (Szczurek et al. 2014). The enlarged insets show that the 2D projection of single molecule fluorophore positions reveals the cylindrical structure of labeled microtubule (diameter = 25 nm microtubule + 10 nm antibodies) - this results in a bimodal distribution of signals along the long axis of the structure (indicated with arrows).

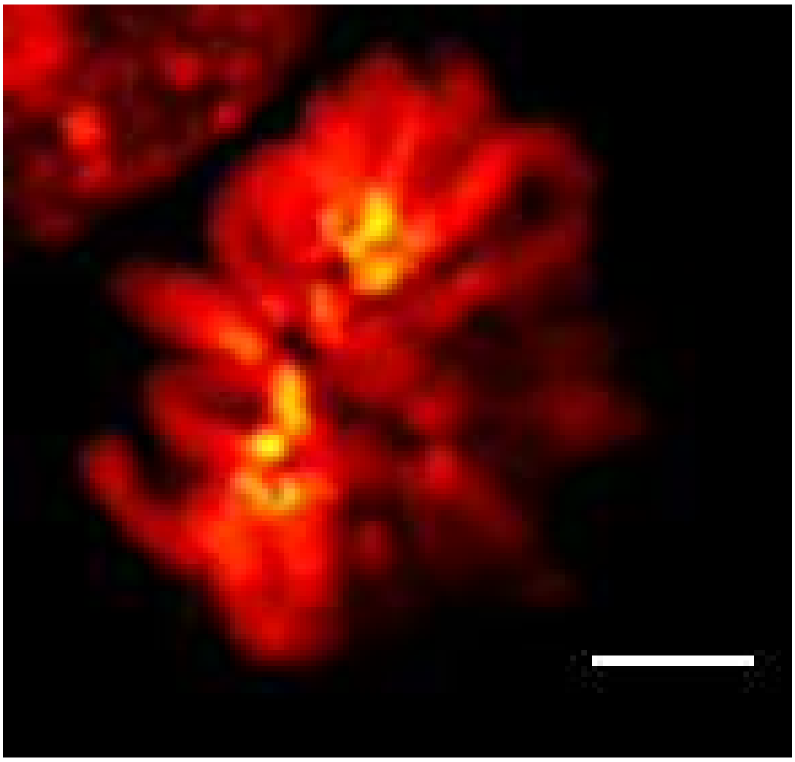

Supplementary Figure S15. Confocal light scanning microscopy (CLSM) of Vybrant dyeCycle Violet stained chromosomes of a dividing cell. CLSM is presently the best among conventional fluorescence microscopy methods. Scale bar corresponds to 2 µm. No mitotic arrest and hypotonic treatment were applied. Part of an interphasic cell nucleus can be seen in left-top corner of the image.

Multicolor DNA/SMLM imaging

A summary/list of fluorescent probes which can be efficiently used in our SMLM approach is shown in Table 2. Particular attention is given to multicolor experiments using fluorescent dyes performing well in conjunction with several DNA dyes suitable for SMLM.

**Fluorophores:**		**Quality of blinking in DNA-buffer**	**Excitation Wavelengths required [nm]:**
**DNA dyes**	**DAPI***	++	405 + 491
	**Hoechst 33258***	+++	405 + 491
	**Hoechst 33342***	+++	405 + 491
	**Vybrant dye cycle Violet®**	+++	491
**Other**	**Alexa 488**	++	491
	**Atto 488**	+++	491
	**Alexa 546**	+	561
	**Alexa 555$**	++	561
	**Alexa 568$**	+++	561
	**Alexa 647***	++++	647 + 491
	**Atto 655**	+++	647
	**Alexa 660***	++	647 + 561
	**Alexa 680***	++	671 + (405 / 491 / 561)

Supplementary Table 2. Qualitative assessment of multicolor SMLM imaging of DNA and other labeled structures. Numerous fluorescent probes are found to perform well in combination with the DNA dyes investigated (number of "+" reflects the quality of performance). For dyes marked with an asterisk it was found that very low intensity illumination at the second (blue-shifted) wavelength effectively increased the number of localized single molecules of fluorophores. *For Alexa 647, Atto 655, and Alexa 660, addition ofl 3 - 5 mM MEA in the buffer improved the blinking with no significant impairment of the performance of VdcV. $ For Alexa 555 and Alexa 568 a protocol of imaging does not necessitate correction of the chromatic shift. We reported on the use of DAPI and Hoechst dyes (photoproducts) in SMLM previously (Szczurek et al., 2014).

References

Baddeley, D., Cannell, M.B., Soeller, C., 2010. Visualization of localization microscopy data. Microsc. Microanal. 16, 64–72. doi:10.1017/S143192760999122X

Benke, A., Manley, S., 2012. Live-cell dSTORM of cellular DNA based on direct DNA labeling. Chembiochem 13, 298–301. doi:10.1002/cbic.201100679

Bernas, T., Zarebski, M., Cook, P.R., Dobrucki, J.W., 2004. Minimizing photobleaching during confocal microscopy of fluorescent probes bound to chromatin: role of anoxia and photon flux. J. Microsc. 215, 281–96. doi:10.1111/j.0022-2720.2004.01377.x

Carlini, L., Manley, S., 2013. Live intracellular super-resolution imaging using site-specific stains. ACS Chem. Biol. 8, 2643–8. doi:10.1021/cb400467x

Cremer, C., Kaufmann, R., Gunkel, M., Pres, S., Weiland, Y., Müller, P., Ruckelshausen, T., Lemmer, P., Geiger, F., Degenhard, S., Wege, C., Lemmermann, N. a W., Holtappels, R., Strickfaden, H., Hausmann, M., 2011. Superresolution imaging of biological nanostructures by spectral precision distance microscopy. Biotechnol. J. 6, 1037–51. doi:10.1002/biot.201100031

Cremer, T., Cremer, M., Hübner, B., Strickfaden, H., Smeets, D., Popken, J., Sterr, M., Markaki, Y., Rippe, K., Cremer, C., 2015. The 4D nucleome: Evidence for a dynamic nuclear landscape based on co-aligned active and inactive nuclear compartments. FEBS Lett. doi:10.1016/j.febslet.2015.05.037

Dempsey, G.T., Vaughan, J.C., Chen, K.H., Bates, M., Zhuang, X., 2011. Evaluation of fluorophores for optimal performance in localization-based super-resolution imaging. Nat. Methods 8, 1027–36. doi:10.1038/nmeth.1768

Heilemann, M., van de Linde, S., Schüttpelz, M., Kasper, R., Seefeldt, B., Mukherjee, A., Tinnefeld, P., Sauer, M., 2008. Subdiffraction-resolution fluorescence imaging with conventional fluorescent probes. Angew. Chem. Int. Ed. Engl. 47, 6172–6. doi:10.1002/anie.200802376

Kaufmann, R., Cremer, C., Gall, J.G., 2012. Superresolution imaging of transcription units on newt lampbrush chromosomes. Chromosome Res. 20, 1009–15. doi:10.1007/s10577-012-9306-z

Kaufmann, R., Lemmer, P., Gunkel, M., Weiland, Y., Müller, P., Hausmann, M., Baddeley, D., Amberger, R., Cremer, C., 2009. SPDM: single molecule superresolution of cellular nanostructures, in: Enderlein, J., Gryczynski, Z.K., Erdmann, R. (Eds.), Proceedings of the SPIE. p. 71850J–71850J–19. doi:10.1117/12.809109

Lemmer, P., Gunkel, M., Baddeley, D., Kaufmann, R., Urich, A., Weiland, Y., Reymann, J., Müller, P., Hausmann, M., Cremer, C., 2008. SPDM: light microscopy with single-molecule resolution at the nanoscale. Appl. Phys. B 93, 1–12. doi:10.1007/s00340-008-3152-x

Reymann, J., Baddeley, D., Gunkel, M., Lemmer, P., Stadter, W., Jegou, T., Rippe, K., Cremer, C., Birk, U., 2008. High-precision structural analysis of subnuclear complexes in fixed and live cells via

spatially modulated illumination (SMI) microscopy. Chromosome Res. 16, 367–82. doi:10.1007/s10577-008-1238-2

Szczurek, A.T., Prakash, K., Lee, H.-K., Zurek-Biesiada, D.J., Best, G., Hagmann, M., Dobrucki, J.W., Cremer, C., Birk, U., 2014. Single molecule localization microscopy of the distribution of chromatin using Hoechst and DAPI fluorescent probes. Nucleus 5. doi:10.4161/nucl.29564

Żurek-Biesiada, D., Kędracka-Krok, S., Dobrucki, J.W., 2013. UV-activated conversion of Hoechst 33258, DAPI, and Vybrant DyeCycle fluorescent dyes into blue-excited, green-emitting protonated forms. Cytometry. A 83, 441–51. doi:10.1002/cyto.a.22260

Żurek-Biesiada, D., Waligórski, P., Dobrucki, J.W., 2014. UV-induced Spectral Shift and Protonation of DNA Fluorescent Dye Hoechst 33258. J. Fluoresc. doi:10.1007/s10895-014-1468-y
